# How the hand has shaped sign languages

**DOI:** 10.1038/s41598-022-15699-1

**Published:** 2022-07-13

**Authors:** Michele Miozzo, Francesca Peressotti

**Affiliations:** 1 Psychology Department, Columbia University, 1190 Amsterdam Av., New York, NY 10027 USA; 2 Dipartimento di Psicologia dello Sviluppo e della Socializzazione, University of Padua, Padua, Italy; 3 Neuroscience Center, University of Padua, Padua, Italy

**Keywords:** Human behaviour, Language

## Abstract

In natural languages, biological constraints push toward cross-linguistic homogeneity while linguistic, cultural, and historical processes promote language diversification. Here, we investigated the effects of these opposing forces on the fingers and thumb configurations (handshapes) used in natural sign languages. We analyzed over 38,000 handshapes from 33 languages. In all languages, the handshape exhibited the same form of adaptation to biological constraints found in tasks for which the hand has naturally evolved (e.g., grasping). These results were not replicated in fingerspelling—another task where the handshape is used—thus revealing a signing-specific adaptation. We also showed that the handshape varies cross-linguistically under the effects of linguistic, cultural, and historical processes. Their effects could thus emerge even without departing from the demands of biological constraints. Handshape’s cross-linguistic variability consists in changes in the frequencies with which the most faithful handshapes to biological constraints appear in individual sign languages.

Natural languages allow humans to communicate an infinite number of ideas accurately and fast. Remarkably, this feat is achieved by organs like the brain, the tongue or the ear not originally designed for language but coopted for it. Biologically, natural languages are subject to the limitations of neuronal computation and of the systems enabling their production and comprehension. Anatomical, physiological, and neuronal constraints have dictated what aspects are permissible in the language, excluding, for example, aspects like speech sounds outside a perceivable acoustic range. The effects of these biological constraints have been proposed to extend to permissible aspects, determining the likelihood with which they appear in a language^1–5^. Aspects optimally satisfying the biological constraints would be favored, while less fitting aspects should be fairly uncommon and likely to be replaced as languages change over time^6^. As the outcome of species-specific evolution and human genetics, biological constraints would impact any language users, and the universality of their effects would push toward cross-linguistic uniformity. But natural languages vary extensively—they are designed to manifest themselves in multiple forms and change due to culture processes and historical events (e.g., migrations, social stratifications). Biological constraints would oppose such variation.

Competing forces are therefore at play—one promoting uniformity, the other variation—and the organization reached by natural languages throughout their evolution corresponds to the equilibrium point of these forces^6–8^. Determining the effects of biological constraints is therefore a critical question not only for explaining why natural languages have emerged in their current form, but also for understanding language variation caused by linguistic, social, and cultural processes. This question was addressed in the present study from the perspective of the sign languages used by millions of signers around the world^9^ that are unique for relying on the fingers and the thumb as primary medium. Humans move the digits (i.e., the fingers and the thumb) with a degree of flexibility and dexterity unparalleled in other primates, conferring them the ability to make very fine movements, powerful grips, and sophisticated interactions with objects^10,11^. In signing, bio-mechanic and functional properties unique to human fingers and the thumb have been coopted for a novel task—to express signs.

Signs are the fundamental units of signing, functionally equivalent to the words of spoken language. Like words, they map onto specific meanings, vary for grammatical class, and change in form to fit into phrases and sentences according to the (morphological, syntactic) rules of a specific language^12^. Signs unfold in space one after the other, and are made either by one hand (usually the dominant one) or two hands^13^ (Fig. 1). Sign can be distinguished one from the other by the movement, orientation, and position of the hand within the signer’s peripersonal space, and by the handshape^14^—i.e., the configuration taken by the fingers and the thumb. In some handshapes, one or more digits are selected by having a different configuration, yet in other handshapes all the digits are given the same configuration^15,16^, as illustrated in Fig. 1. The fingers and the thumb vary in a similar way in manual activities of everyday life, moving independently or in synchronized fashion, and taking configurations shared either by some or all digits. Investigations in movement science that examined the selection of an individual digit and the coordination of multiple digits have shed light on the anatomical and neuronal constraints underlying the selection of one and multiple digits^17,18^. Their findings motivated our choice of focusing here on the handshape. We therefore examined to what extent the handshape conforms to the constraints identified in movement science.

**Figure 1:**
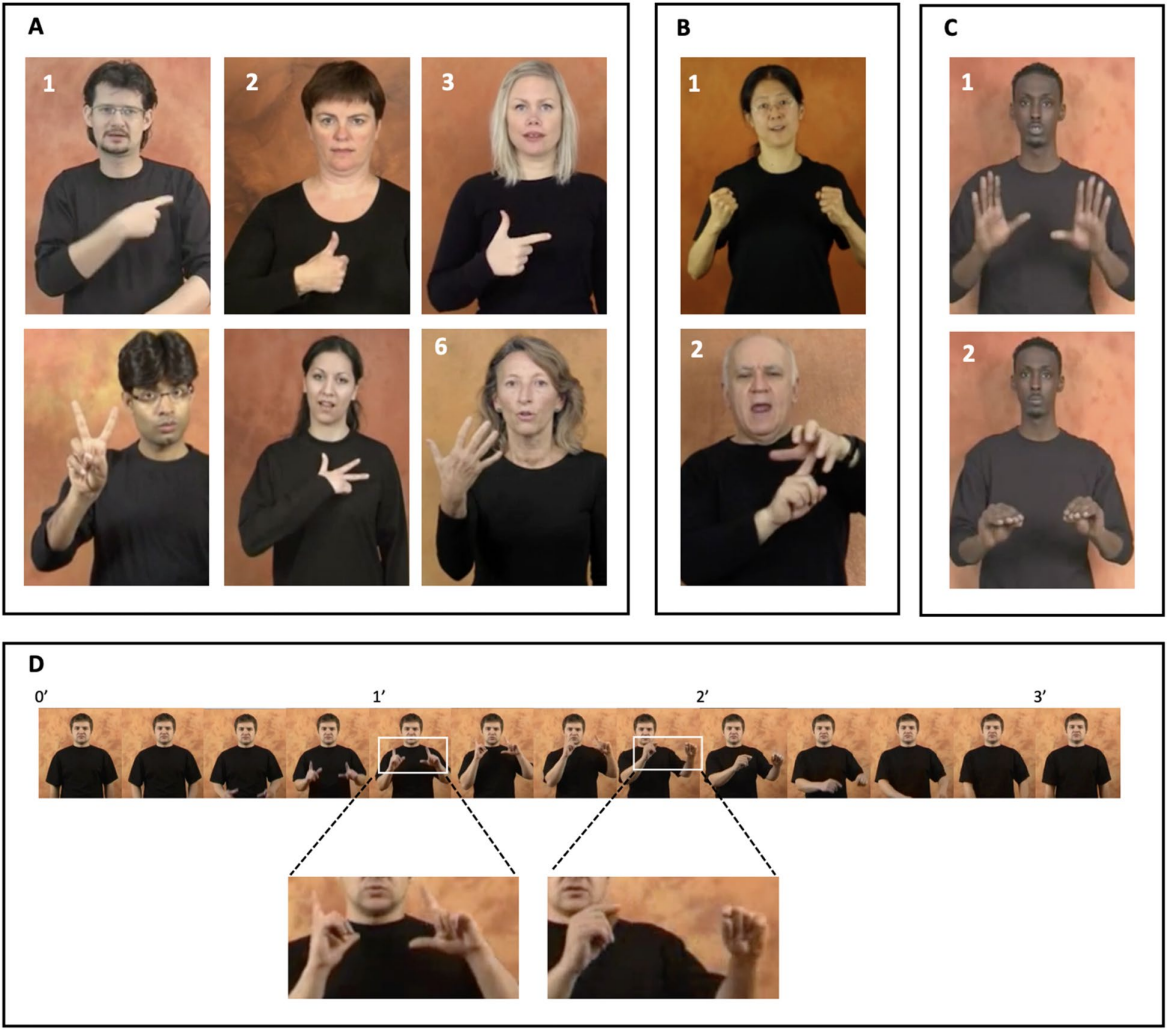
The signs in Panel (**A**) were made by the dominant hand and vary for handshape: in (1)–(5) the thumb or one or more fingers differ in shape from the other digits; in (6) the four fingers are identically shaped. The signs in Panel (**B**) were made by both hands; dominant and non-dominant hands are shaped identically (1) or differently (2). The sign in Panel (**C**) includes two handshapes, the first occurring at the beginning of the sign, the second at the end of it. Panel (**D**): frame-by-frame view of one of the videos we analyzed. The video showed one sign, and started and ended with the signer in resting position. Numbers correspond to recording time (in s). Enlarged pictures show the two configurations scored for this sign. Pictures of the signs are from the website *Spread the Sign*^55^.

Control of individual digits was examined in laboratory studies by instructing participants to move a specific digit^19,20^ or to apply force with only one digit^21,22^. Movement and force were not confined to the instructed digit but were observed in other digits too, although with appreciable differences among digits. The thumb showed the greatest autonomy, followed—in this order—by the index, little, middle, and ring fingers^19^. Variation in digit individuation has been explained as reflecting the anatomical and neural organization of the human hand^17,23,24^. For example, the thumb owns its autonomy to a distinct musculature^25^ and a distinct cortical representation in primary motor cortex (M1)^26^. Anatomical structures that mechanically couple the fingers include the soft tissue in the web space between the fingers, the interconnections between the tendons of the extrinsic muscles, and the muscle bellies of the extrinsic muscles^24^. The mechanical and neuronal features limiting finger autonomy facilitate finger coupling, though with graded effects across fingers. Kinematics and neurophysiological studies have shown that immediately neighboring fingers are most closely correlated^24,27,28^. In a post-mortem investigation of the human hand, traction to the extensor tendon of any given finger resulted in the extension of all fingers, although the effect was scaled, depending on proximity to the finger to which the traction was applied^29^. The same graded pattern emerged using an in vivo technique designed to isolate the contribution of mechanical and neural sources, respectively^24^. Further evidence suggesting a neural source was found by recording the EMG activity of the flexor digitorum profundus (FDP), the only muscle attaching to the distal phalanx of each of the four fingers^30^. In several compartments of the FDP, activity appeared not only during the flexion of a specific finger, but also during the flexion of its immediate neighbor finger. Furthermore, in multiple extrinsic muscles, the strongest short-term synchronous firing of two motor units appeared in neighboring digits^28^.

The easiest configurations to program and execute on mechanical and neural grounds are those in which, like in grasping, all fingers, moving simultaneously, take an identical shape^31^. The simultaneous movement and identical shaping of all fingers is facilitated by the presence of anatomical structures mechanically coupling the fingers^23^. Controlling each of the 15 joints forming the hand, which collectively afford approximately 20 degrees of freedom^24,31^, is computationally demanding. Configurations formed by identically shaped fingers simplify neural processing, consistent with data from kinematic analyses, contact forces, electromyography (EMG)^23,32^, and naturalistic recordings during everyday activities^33^ showing that coordinated patterns (synergies) underlie the synchronous movements of all fingers with no needs to independently control each joint.

Collectively, the findings from movement science have revealed that the digit configurations optimally satisfying the mechanic and neuronal constraints in tasks for which the hand has naturally evolved are the ones in which (a) the one selected digit is either the thumb or the index, (b) neighboring fingers are coupled, and (c) all fingers are identically shaped. The same preferences should be observed with the handshape, if mechanic and neuronal constraints have similar effects here. We tested this hypothesis by examining the handshapes of over 28,000 signs in 33 languages, focusing on the frequencies with which different types of handshapes occurred. Constraints derived from human genetics and the species-specific evolution of the hand are a vehicle for cross-language uniformity. As another means to assess the strength of the constraints, we examined the similarity existing among handshapes from geographically distant and historically unrelated languages^34,35^. Linguistic theories have explained various aspects of sign languages in terms of digit control^12,13,15,16,36,37^. Our investigation, however, departs from prior linguistic studies in two ways. First, data on digit control in non-linguistic tasks were used as predictors of the handshape. Second, we analyzed a far larger number of languages—our corpus of 33 languages is the largest to date.

The handshape could demand a larger use of less conforming configurations, thus showing a lesser degree of adaptation to mechanic and neuronal constraints than the one observed with non-linguistic tasks. One possible reason for its weaker adaptation is that the cross-linguistic uniformity resulting from biological constraints could contrast cross-linguistic variation. Clear and successful communication also depends on variability—a too small handshape vocabulary might not allow building the sign languages presently used. This might be especially limiting for sign languages, which make extensive use of iconicity^38,39^. In many signs, hands are shaped, moved or positioned to visualize key properties of the concepts they represent, as with the sign bike produced in many languages by circling the hands shaped as fists. If handshapes conforming more closely to biological constraints could not be iconic, iconicity would have driven the selection of less faithful handshapes. But it is also possible that some biological constraints were overcome in sign language. Extensive hand use in a task as demanding as signing could have weakened some of the effects of mechanic and neuronal constraints, as has been found with piano players who demonstrated greater finger individuation^40,41^. Furthermore, sign languages are typically acquired at a later age relative to spoken languages, because most of deaf infants have hearing parents and are therefore exposed late to signs^42^. Limitations that would have made complex handshapes difficult to the younger child, would be no longer present at an older age, so that the effects of those limitations could be tempered with the handshape. Despite these plausible reasons for lessening the biological constraints, it is equally possible that these constraints are not bendable, so that the adaptation of the handshape would not differ from the one found with non-linguistic tasks. An objective of our study is to clarify the bendability of the constraints.

Establishing whether adaptation differs between the handshape and non-linguistic tasks has implications also for characterizing the language variation originating from linguistic, social, and cultural processes. Our data would help us understand if lessening the biological constraints is a necessary condition for the emergence of language variation driven by these processes. Despite their contrasting effects, these opposing forces may not always compete for the same resources. With respect to sign language, this means that linguistic, social, and cultural processes would affect other aspects than the handshape. A crucial challenge we face in understanding the interaction of these opposing forces is therefore to show that the handshape varies as an effect of linguistic, social, and cultural processes. The cross-linguistic approach of our study provided an opportunity to tackle this issue. The sign languages we examined have been grouped in distinct language families based on their history and linguistic characteristics^34,35^. We examined if handshapes varied systematically across these language families, and considered this type of finding as showing that handshapes change under the pressure of linguistic, social, and cultural processes.

Different types of analyses were conducted on the handshapes from 33 sign languages to address the questions examined in our study. The extent to which the handshape conforms to the biological constraints was investigated in two ways. First, we examined if the finger configurations favored in non-linguistic tasks were preferred to the same degree in the 33 languages. Second, we investigated if individual handshapes occurred in similar frequencies across languages, thus conforming to the cross-linguistic consistency expected from the biological constraints. To investigate whether the biological constraints underlying the handshape are bendable, we turned to another linguistic system using the handshape—fingerspelling. We therefore analyzed the extent to which the finger configurations favored in non-linguistic tasks were also preferred in fingerspelling. Finally, to establish whether handshape cross-linguistic variability resulted from linguistic, social, and cultural processes we attempted to reconstruct the language families of the 33 sign languages from handshape data.

## Results

### Digit selection in signing

Research on the movement of the fingers and the thumb in non-linguistic tasks has revealed aspects in the selection of one, two, and multiple digits that reflected mechanical and neural constraints^17,18^. We examined if these aspects appeared in signing and therefore the frequencies with which digits were selected in sign languages mirrored findings from the movement of the fingers and the thumb in non-linguistic contexts. Analyses were based on the percentages with which handshapes occurred in the 33 languages (*total percentages*) or in each of these languages (*language percentages*).

**Figure 2:**
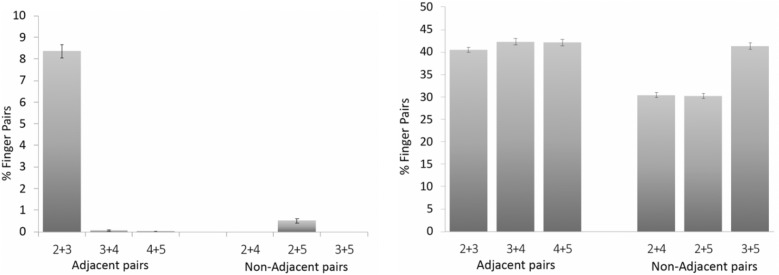
Percentages of handshapes in which two fingers were selected (left) or identically shaped (right) in the sign. Adjacent pairs included neighboring fingers; Non-adjacent pairs were formed by distant fingers. Two fingers were more likely to be selected or identically shaped if they were immediate neighbors (ps < 0.001). 2 = index finger, 3 = middle finger, 4 = ring finger, 5 = little finger.

- *One digit selection *The various measures that have been used to determine how independently each digit moves revealed a precise hierarchy^19–24^. The thumb was the most autonomous, followed by the index, little, middle, and ring fingers. We examined if the same hierarchy appeared in handshapes in which a digit was selected. When selected, the thumb or a finger was shaped differently from the other four digits for being, for example, relatively more flexed or extended (Fig. 1A). We calculated an independence score for each digit in the handshape, which corresponded to the total percentage of handshapes in which the digit was selected—i.e., it was shaped differently relative to the other four digits. Independence scores revealed the same hierarchy observed in studies of digit movement: the thumb was the most selected (28.7%), followed by the index finger (12.6%), middle and short fingers came next (0.8%), the ring finger last (0.03%). This ranking appeared in the 33 languages (data are reported in SI A4). Correlations were used to determine how closely digit selection in the handshape mirrored the independence with which digits moved. Specifically, our independence scores were correlated with four measures of digit individuation available from published studies of hand movement^19,33^. Although we did not analyze how digits moved to generate the handshapes, movement measures represented a suitable proxy, as handshapes were typically realized by moving the selected digit. Two of the measures we used were from Häger-Ross and Schieber^19^. They were obtained by instructing participants to move one digit a time, and quantified the degree to which the non-instructed digits either moved (individuation index) or remained still (stationarity index). The other two measures, from Ingram et al.^33^, were based on hand movements in everyday activities; one corresponded to the percentage of variance that in a linear reconstruction of digit angular velocities was unexplained by the movements of the other four digits; the other was the percentage of time during which a digit moved independently. Independence scores derived from handshapes were strongly correlated with each of these measures (mean r = 0.916)—in fact, as high as among these measures themselves (mean r = 0.913; Table 1).
- *Digit pairing *Kinematics and neurophysiological studies have shown that immediately neighboring fingers were most closely correlated^24,27,28^. In signing, this topography of finger interaction would have tended to favor the coupling of adjacent fingers. In two tests of this prediction, pairs of neighboring fingers were compared to pairs of distant fingers, and language percentages were analyzed (language percentages are reported in SI A5). The first test examined if both fingers in the pair were selected—i.e., they were shaped differently than the non-selected fingers and the thumb (Fig. 1A). Two fingers were more often selected together if adjacent (means 2.8% vs. 0.2%; t(32) = 23.48, p < 0.001; Fig. 2). The second test analyzed if the two fingers in the pair were identically shaped—e.g., they were both closed or curved. This occurred more often with neighboring than distant fingers (means 41.6% vs. 33.9%; t(32) = 55.69, p < 0.001; SI A5; Fig. 2).

**Table 1:** Correlation coefficients (rs) of correlations between different measures of digit individuation.

	Independence score/signing	Individuation index^19^	Stationary index^19^	% Unexplained variance^33^	Movement Time^33^
Individuation index^19^	0.821				
Stationary index^19^	0.903	0.953			
% Unexplained variance^33^	0.998	0.835	0.924		
Movement time^33^	0.943	0.845	0.954	0.963	
Independence score/fingerspelling	0.411	0.770	0.557	0.408	0.356

Data on finger coupling were obtained in Ingram et al.^33^ by recording finger movements in daily activities. The time during which, in all pairs, the two fingers moved together in Ingram et al.^33^ was correlated with the percentages of handshapes in which, in all pairs, the two fingers were identically shaped. The two measures were strongly correlated in all languages (mean r = 0.865; range = 0.654–0.911).

Handshapes in which a finger and the thumb were both selected were quite common, with a total percentage of 26.9%. The movement-time analyses of Ingram et al.^33^ revealed that the thumb is more likely to be paired exclusively with the index finger rather than any other finger. A similar preference appeared in our corpus: the thumb was paired mostly with the index finger (87.2%). In all handshapes in which the thumb and the index finger were selected, the non-selected fingers (middle, ring, and little) were identically shaped, a finding showing strong coupling among these three fingers. In these handshapes, the middle, ring, and little fingers could have functioned in synergy as a unit, a profitable feature in light of results showing that these are the most strongly coupled fingers^28^.

(iii) *Multiple-digit selection *The analyses of the handshapes requiring multiple-digit selection were of two kinds. The first type of analysis concerned the incidence of handshapes in which the four fingers were identically shaped. They represented the most common type of handshape in each of the 33 languages (mean 55.9%; range 47.3–63.2%), a result aligning with the movement-time analyses of Ingram et al.^33^ that showed that the configurations in which the fingers moved in synergy were the most frequent in daily activities. In the second type of analysis, handshapes were compared to grasping, a movement requiring multiple-digit selection and coordination that has been extensively investigated^43^. A distinction has been drawn between power and precision grasps on kinematic, phylogenetic, and ontogenetic grounds^43–46^. In power grasps, which are evolutionary more primitive and appear earlier in human development, all digits are flexed around the object to provide high stability. An example of power grasp is handshaking. Precision grasps, used for manipulating small objects, require independent finger movements and fine control—as when plucking a flower that the thumb and the index finger are placed opposite to each. We identified 13 handshapes with the same digit configurations of the grasps listed in the GRASP Taxonomy^47^, a comprehensive classification of every static hand posture that allows holding an object securely with one hand. (Grasp-like handshapes are shown in SI A6.) Handshapes replicating the configurations of power grasps were much more common across languages relative to those that were similar to precision grasps (means 13.4% vs. 4.3%; t(32) = 25.89, p < 0.001; data reported in SI A7), a finding revealing a preference for signs requiring less digit individuation and hand control^31^. The relation between grasp-like handshapes and the corresponding grasps was further investigated by examining the correlation between the language percentages of these handshapes and the frequency and duration of the corresponding grasps in daily activities reported in Bullock et al.^48,49^ Strong correlations were found with frequency (mean r = 0.715; range 0.584–0.866) and duration (mean r = 0.817; range 0.621–0.915).

### Digit selection in fingerspelling

It is possible that the strong correlations observed with the handshape would emerge with any tasks requiring fine motor control. To address this question, we turned to fingerspelling, a linguistic task in which a manual alphabet is used to represent the letters of a writing system, typically when sign equivalents are lacking. The manual alphabets presently used in signer communities across the world vary by employing one hand or two hands, and in representing different alphabets (e.g., Latin or Arabic)^35^. Despite their differences, the handshape is a fundamental component of all alphabets. To produce the letters forming the words, fingers are thus selected and shaped with precision and speed comparable to signing. Notably, some handshapes are used in both signing and fingerspelling. The terms *letter handshapes* and *sign handshapes* were used to refer to the handshapes observed in fingerspelling and signing, respectively.

We scored the letter handshapes (n = 1947) of 63 manual alphabets from different writing systems (Latin, Cyrillic, Greek, Arabic, Hebrew, or Korean), using the same method employed with sign handshapes (manual alphabets and sources are listed in SI A8). Of the 108 distinct handshapes identified among letters, 90.7% were also found among signs—a result further confirming the similarities of the digit configurations used in fingerspelling and signing. The analyses on the selection of one, two and multiple digits conducted with sign handshapes were replicated with letter handshapes. Similarities with respect to non-language tasks were found with finger pairing. Compared to two distant fingers, two immediately neighboring fingers were more likely to be selected together (9.2% vs. 0.9%; χ^2^(1) = 416.10, p < 0.001) or identically shaped (72.3% vs. 39.1%; χ^2^ = 1307.42, p < 0.001). Furthermore, the percentages of letter handshapes in which the two fingers were identically shaped correlated strongly (r = 0.974) with the time during which the two fingers moved together in Ingram et al.^33^ Differences appeared, however, with handshapes requiring one-digit selection. Independence scores were calculated for each digit in the letter handshapes. Independence scores correlated relatively weakly with the measures of digit individuation obtained in non-linguistic tasks (Table 1). These weak correlations reflected major differences in digit individuation ranking. While the thumb was the most independent digit in non-linguistic tasks, the index finger was the most selected digit in fingerspelling (16.9%); furthermore, the thumb was selected as frequently as the little finger (6.0% vs. 6.1%). Additional differences emerged with grasp-like handshapes. Not only they were less frequent among letter handshapes than sign handshapes (8.2% vs. 23.1%; χ^2^(1) = 236.17, p < 0.001), but they also correlated less strongly with the frequency (r = 0.423 vs. 0.715) and duration (r = 0.624 vs. 0.817) of the corresponding grasps in daily activities^48,49^.

Overall, the findings from fingerspelling indicated that the strong correlations that emerged with sign handshapes are not reproducible in some manual tasks, suggesting that the effects of mechanic and neural constraints can be weakened.

### Cross-linguistic variation

**Figure 3:**
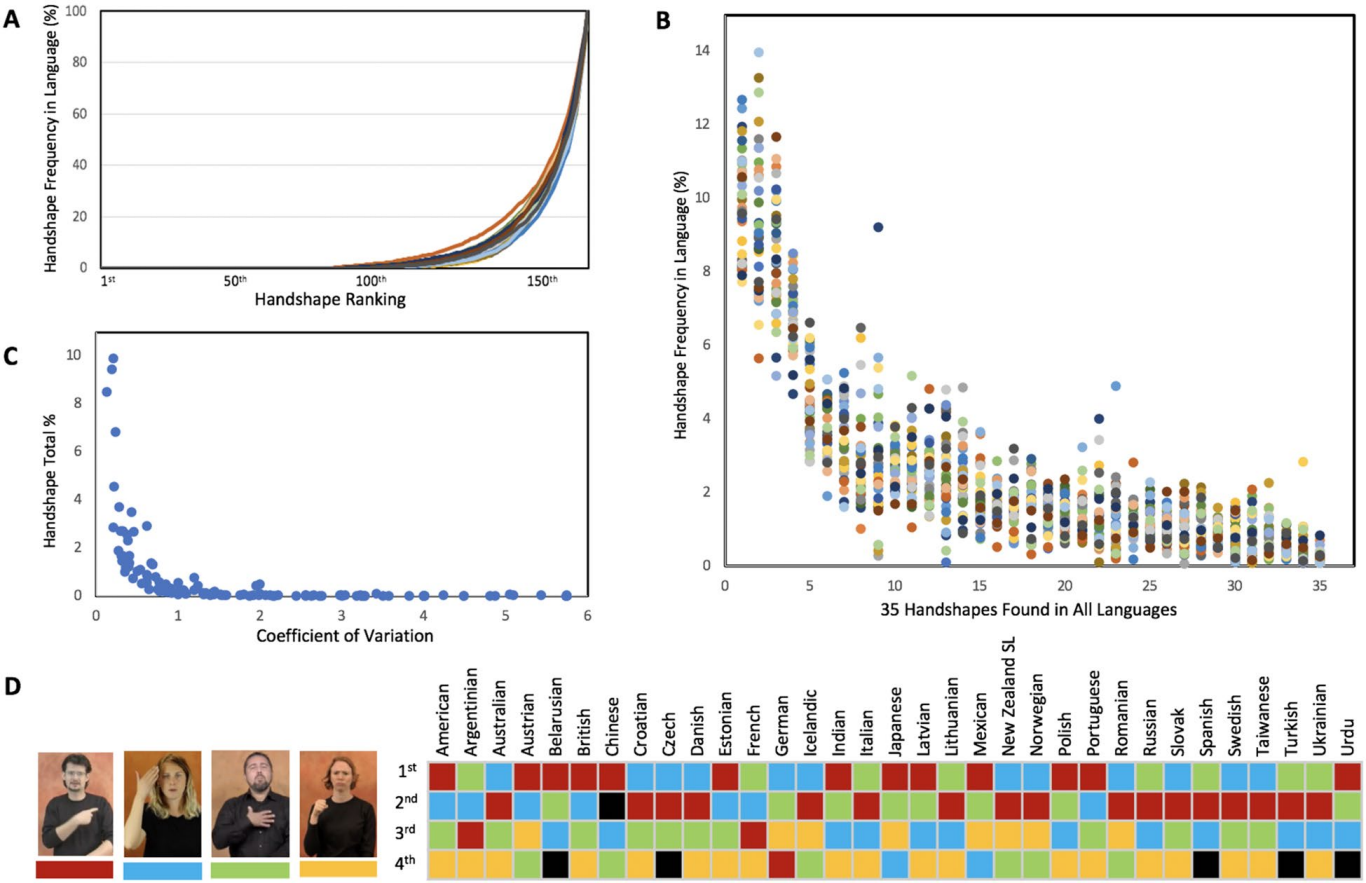
(**A**) Each line shows the cumulative frequencies for the handshapes in one of the 33 languages. The 160 handshapes found in the 33 languages are ordered on the x axis according to their ranking in each language. The 160th handshape is always the most frequent; the specific handshape on each ranked position may vary from one language to another. Cumulative frequencies were similarly distributed across languages. (**B**) Frequencies of the handshapes (n = 35) observed in all of the 33 languages. Each column shows one handshape; the dots in a column correspond to the frequency of the handshape in the 33 languages. Handshapes had frequencies that patterned similarly across languages. (**C**) Coefficient of variation (a standardized measure of dispersion) was inversely correlated (r = − 0.434, p < 0.001) with the percentages with which handshapes occurred in the 33 languages (Handshape Total %). (**D**) Colors indicate which of these handshapes was among the four most frequent handshapes in each language. With only 6 exceptions (colored in black) these handshapes ranked top four. Pictures of the signs are from the website *Spread the Sign*^55^.

**Figure 4:**
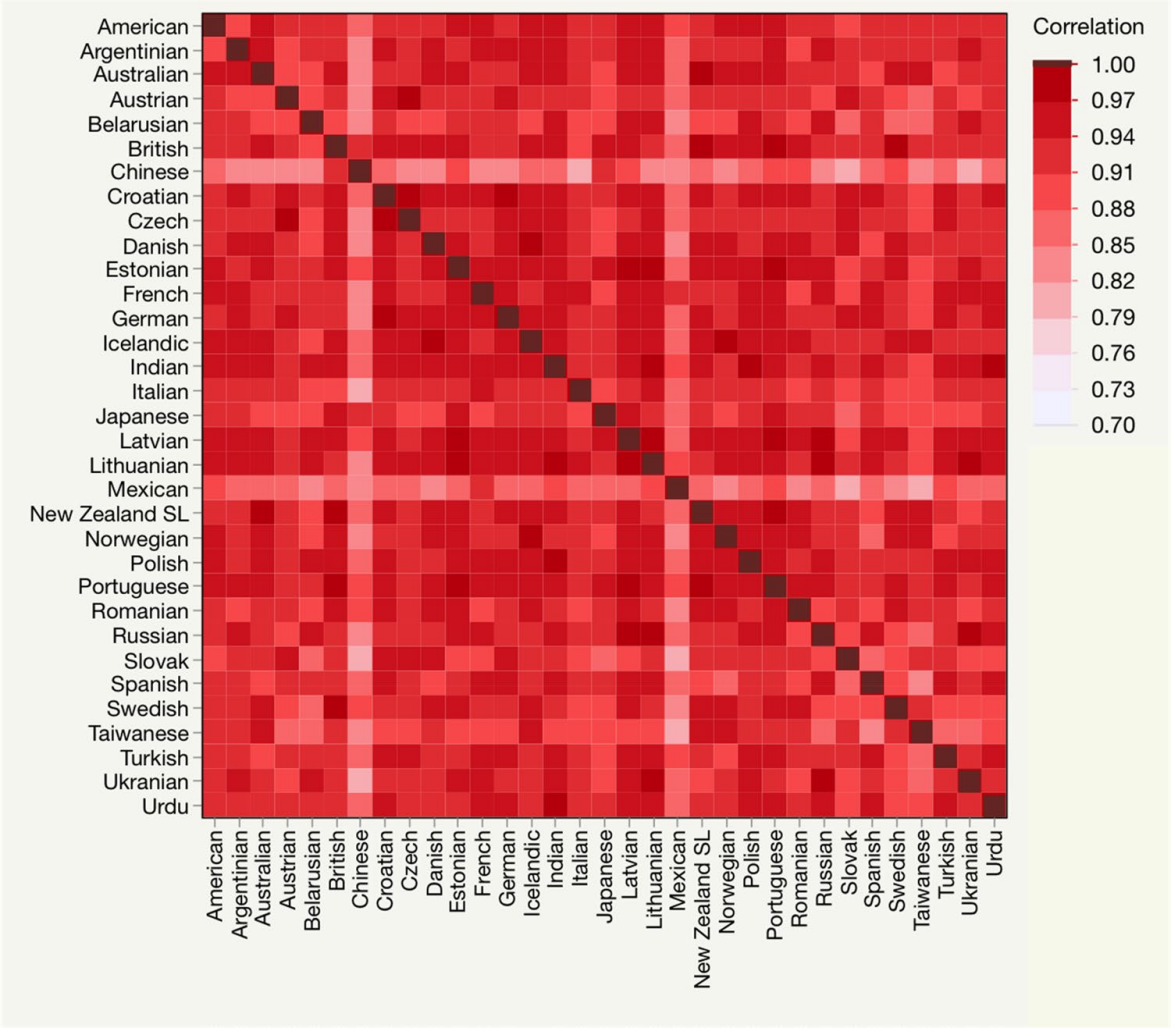
The heat map shows the correlation coefficients resulting from correlating the frequencies of the 160 handshapes between each language pair. Handshape frequencies were strongly correlated across languages (mean r = 0.925; range 0.799–0.986).

Language statistics can provide further evidence on the effects of biological constraints underpinning the handshape. Biological constraints are expected to have similar effects across the 33 languages we examined. In this respect, handshapes showed marked similarities. First, cumulative frequencies patterned very similarly and revealed the predominance, in every language, of a few handshapes (Fig. 3A). Second, 35 common handshapes were found in all 33 languages; their total percentage was equal to 89.2%, and they were distributed very similarly cross-linguistically (Fig. 3B). Furthermore, the same handshapes were the most frequent in almost any of the languages (Fig. 3D). Third, language percentages were strongly correlated between any of the language pairs (mean r = 0.925; range 0.799–0.986; Fig. 4). These correlations remained equally strong in analyses restricted to the 35 common handshapes that we conducted to rule out that the high correlations resulted from the absence of some handshapes in most languages (mean r = 0.891; range 0.651–0.980).

Handshapes most faithful to the mechanic and neural constraints included those in which the fingers were identically shaped, and the most independent digits (thumb and index finger) selected. They represented 85.6% of the handshapes in the 33 languages, and corresponded to 31/35 common handshapes. Conformity to those constraints would have made the most frequently occurring handshapes less permeable to linguistic and cultural pressure specific to each language, and consequently less likely to vary across languages. Pressure—and variance—would be evident especially with less frequent handshapes. In other words, handshape frequency and handshape variance would be negatively correlated. The degree to which a handshape varied (in percentage) across languages was estimated using the coefficient of variation, a standardized measure of dispersion corresponding to the ratio of standard deviation to the mean. Coefficients of variation were negatively correlated with total percentages (r = − 0.434, p < 0.001; Fig. 3C; data are reported in SI A10), thus revealing that variability increased as handshape frequencies decreased.

### Language families

A hierarchical cluster analysis was conducted on the handshape frequencies in the 33 sign languages to determine if modern sign languages grouped in ways that reflected their history and cultural similarity. Our goal was not to reconstruct the evolution of sign languages nor to map the chronology of language changes, goals that have been pursued in computational historical linguistics by employing other statistical tools than the hierarchical cluster analysis^50^. Rather, as a technique for dividing a dataset into groups of similar objects^51^, the hierarchical cluster analysis suited our specific goal of determining whether similarities in the cross-linguistic variation of handshapes mirror historical and cultural relations.

**Figure 5:**
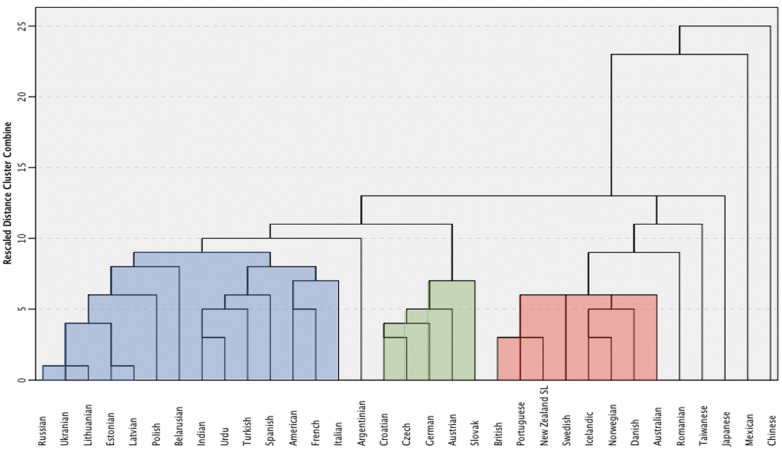
The dendrogram illustrates the three language families revealed by the hierarchical cluster analysis that was conducted on the handshape frequencies of 33 languages. Languages were historically related to French (blue) or Northern-European languages (red), or they were part of the central-European group (green).

Although the development of individual sign languages has not been fully mapped out, historical circumstances and geographical proximity appear to be crucial^34,35^. In some historical contexts, for example, signers from other countries were instrumental in exposing local deaf communities to sign languages, a type of language contact that happened between sign languages like French and American^52^, Italian and Argentinian^53^, or Swedish and Portuguese^34^. It has been proposed, on linguistic and historical grounds, that most of the languages we analyzed belong to one of these three language groups: one formed by languages historically tied to French, one grouping central European languages, and one related to Northern European languages^34,35^. Chinese, Taiwanese, and Japanese sign languages evolved independently of these language families^34^. The three major groups that emerged from the hierarchical cluster analysis correspond to each of these language families, as shown in Fig. 5. Within each group, the more similar languages tended to be historically, geographically, or culturally related. For example, Russian sign language was similar to sign languages used in nearby countries (e.g., Ukraine or Lithuania). Other languages (e.g., Taiwanese and Japanese) were more weakly associated with these families, reflecting their relative isolation^34^. The hierarchical cluster analysis also revealed similarities that do not seem to be historically based. Indian, Urdu, and Turkish sign languages are not historically linked to sign languages related to the French sign language^54,55,56^, nor there is evidence associating Japanese sign language to the sign languages of the Northern European family^34^. While these incongruencies show that our hierarchical cluster analysis did not provide a comprehensive historical reconstruction, they further suggest that our analysis detected similarities in the cross-linguistic distribution of handshapes not rooted in language history.

## General discussion

An adaptive strategy to deal with the speed and precision with which the fingers and the thumb are moved and positioned in signing would likely favor those handshapes that can be easily articulated. Our results revealed that these are indeed the handshapes preferred across languages. But they also showed that these preferences closely mirrored those observed in non-linguistic tasks, which in turn reflected mechanical and neural constraints^17,18,31^. In both cases, the most independent digits tended to be selected, the immediate neighbor fingers tended to be paired, and the most common configurations were those in which all fingers were identically shaped. Furthermore, measures of digit independence and finger coupling obtained in non-linguistic tasks proved to be reliable predictors of the handshapes observed in 33 languages. The handshape thus demonstrates an adaptation to biological constraints that is remarkably similar to that found in tasks for which the hand naturally evolved. This strong similarity appears not because other forms of adaptation cannot arise—in fingerspelling, the handshape deviates from those constraints. The handshape’s adaptation seems to be a potentially favorable outcome rather than an inevitable one.

Even if not based on movement, our results overlapped consistently with results from digit movement. These strong similarities could have emerged because we analyzed categorical descriptions inherently associated with the movements made by the joints to bring the fingers and the thumb into their positions in the handshape. For example, similarities with handshapes in which the thumb was selected would have resulted from moving the thumb. We should also observe that digit movements are organized along properties like digit selection or finger coupling upon which the system we used for handshape categorization^52^ is based. As we specifically examined these properties, digit-movement data were naturally suited to unveil similarities with the handshape.

Beside large commonalities, there were also two noticeable discrepancies between signing and the data on natural hand movement of Ingram et al.^33^. In signing, the four fingers formed synergetic patterns more frequently (60% vs. 42%) and individual digits were selected more often (38% vs. 17%). Both differences reflected an increase of faithful digit configurations. Configurations with identically shaped fingers are computationally the least demanding^31^, while the increase in digit individuation resulted from a larger use of the thumb and the index finger, the most independent digits^22,24^. These findings show that there are instances in which the handshape conforms to biological constraints even more strongly than non-linguistic tasks.

The scale of our study allowed exploring the statistics of the handshape. Cross-linguistically, handshapes were very similar in terms of frequencies and distribution (Figs. 3, 4). These statistical properties are the foreseeable outcomes of biological constraints. Strong cross-linguistic similarities are indeed expected from constraints reflecting human genetics and the evolution of the human hand that are universal. Biological constraints would also drive the high incidence in the language of the most faithful handshapes. They indeed accounted for most of the handshapes in 33 languages (85.6%) and most of the types of handshapes observed in all of those languages (31/35). And as expected by the effects of biological constraints, the frequencies of the most faithful handshapes were highly consistent across languages (Fig. 3C).

The strong cross-linguistic similarity we observed has not prevented the handshape to diversify in ways determined by their linguistic, historical, and cultural specificity^34,35^. The linguistic families we were able to reconstruct from the handshape (Fig. 5) highlight the contribution of linguistic, social, and cultural processes to determining what handshapes surface in each language. The cross-linguistic variation driven by these processes co-exits with the handshape’s close adaptation to biological constraints. This has likely limited the type of cross-linguistic variation that could emerge with the handshape. The strict conformity to biological constraints has probably precluded forms of language diversification that involve selecting the less faithful handshapes or selecting only some of the favored handshapes in a given language. A more viable option would be to maximize the presence of the most faithful handshapes while changing slightly the incidence of each of these handshapes across languages—a form of language variation that is distributional and statistical in nature.

Less faithful handshapes are rather common in fingerspelling. It is perhaps not a coincidence that the handshape conforms to the biological constraints less in fingerspelling—a product of culture and explicit learning—than in natural sign languages. As the elements of a codified writing system acquired in institutionalized forms, manual letters are standardized and difficult to change. By contrast, being part of natural languages, signs are more malleable, as described, for example, with iconicity. Several examples have been documented of signs that were originally iconic and then became increasingly more abstract^53^. The same process has not been observed with manual letters, whose iconicity persists^35^. There is certainly an advantage to preserve their iconicity, as it would facilitate their recognition^53^. But what was gained in recognition was lost in motor control.

The four most common handshapes, shown in Fig. 3D, amounted collectively to 34.7% of the handshapes we analyzed. Their astounding frequency seems justifiable from a motor-control perspective—index finger selection and identical finger configuration make them maximally conforming to biological constraints. However, limiting the handshape repertoire to these four handshapes would have impacted the efficacy of communication. Sign languages tolerate handshapes of such frequencies, because signs can also be distinguished by the movement, position, and orientation of the hand^14^. To the extent that sign multidimensionality ensures a rich lexical diversification, handshapes closely conforming to biological constraints could emerge even in very high frequencies. Linguistic contexts in which conformity to biological constraints would compromise communication efficacy would be less permeable to those constraints. This could be one of the reasons for which fingerspelling conforms less to biological constraints. Its more limited use of movement, position, and orientation and its greater reliance on the handshape^35^ could have in part determined its reduced conformity.

As Ejaz et al.^26^ reported in their neuroimaging study, the spatial organization of finger-specific activity in M1 was predicted by the statistics of natural hand movement from Ingram et al.^33^ That the same statistics correlated closely with our data would lead one to speculate that the same cortical organization is recruited for signing. Although this conclusion needs empirical corroboration, it is worth highlighting that the functional similarities we found between signing and hand movement would make a shared cortical representation more plausible. Developmentally, a shared representation would yield some advantages. First, because hand movement and signing would reinforce the development of the same cortical organization. Second, because it would temper the effect of delayed acquisition of signing. Most deaf children do not have signing parents and are therefore exposed to signing at later age than that at which their hearing peers are exposed to spoken language^42^. This delay would be less consequential if the cortical organization initially shaped by hand usage is later shared with signing—which could represent another reason for the handshape’s strong adaption to the biological constraints.

In conclusion, by analyzing 33 sign languages, we showed that handshapes exhibit the same form of adaptation to biological constraints found in tasks for which the hand has naturally evolved, and found considerably high consistency across sign languages in the use of handshapes closely fitting biological constraints. Nonetheless, such stringent biological constraints have not prevented sign languages to differentiate among each other as a consequence of linguistic, cultural, and historical processes.

## Methods

We selected 800 concepts that varied for semantic field and concreteness/abstractness, and were represented by words and signs from several grammatical categories (nouns, verbs, adjectives, adverbs). We conducted a search of online sign language dictionaries to identify sign languages with > 85% of those concepts available, a criterion met by 33 languages (22 European, 6 Asian, 3 American, 2 Australasian). Signs were found, across languages, for an average of 97.6% concepts (range 87.3–100%). If multiple signs were listed in a dictionary entry, all variants were included. We excluded 221 signs, because a word was fingerspelled (54%), a sentence was used to describe the concept (39%), or the sign was not perfectly visible on the video (6%). 28,343 signs were analyzed, an average of 859 signs per language (range 677–1192).

Online dictionaries showed the sign corresponding to one concept only per video. Some signs were compounds formed by two or more signs, the equivalent of the word *football* or *blackboard*. Most of the signs were produced only by the dominant hand (49.0%; Fig. 1A); in 2-hand signs, the two hands were shaped identically (30.6%; Fig. 1B1) or differently (20.4%; Fig. 1B2). We analyzed the handshapes of the dominant hand, because it articulates more handshapes and its handshapes are of greater variety and complexity^13,37^. Within a sign, the dominant hand produced one handshape (76.4%; Fig. 1A) or two handshapes (23.6%; Fig. 1C). Before beginning to sign, signers stood in front of the camera with the hands in resting position. Digit configuration changed continuously between resting position and the fully formed handshape, or while moving from one handshape to the next in signs with multiple handshapes. We only analyzed the configuration of the handshape, not configurations prior to it. Signs have typically only one movement^12^, which provides clear spatial and temporal boundaries of the sign, as can be seen in the examples in Fig. 1D. All video-recordings could be viewed at reduced speed. Handshapes were classified according to the Hamburg Notation System for Sign Languages^57^ that was developed to describe all handshapes from any language. The transcription obtained for each handshape specified if the fingers and the thumb were selected or spread, as well as their shape (extended, bent, flattened, hook, or closed). The thumb or a finger was classified as selected if it was shaped differently from the other digits. Orientation was ignored, so were short, fast, repetitive movements such as wiggling. Handshapes that were repeated within a sign were only scored once. Two people with knowledge of a sign language independently scored the handshapes. Discrepancies were rare (0.1%) and consistently resolved. The signs we analyzed were formed by 38,035 handshapes performed with the dominant hand, an average of 1153 handshapes per language (range 870–1505). Within this corpus of handshapes, we identified 160 distinct configurations using the classification system described above. Only 35/160 (21.8%) of distinct configurations were found in all languages. We analyzed the percentages with which handshapes occurred in the 33 languages (*total percentages*) or in each of these languages (*language percentages*). Signs, web sources, and data on individual languages are reported in Supplementary Information A1–A3; the distinct configurations are shown in Supplementary Information B. Handshapes were also classified by number of selected digits (Fig. 1A). A digit was scored as selected if it differed in shape from the other four digits. When one digit was selected, it was either a finger or the thumb, whereas the two selected digits were either two fingers, or a finger and the thumb. In three-selected-digit handshapes, the selected digits (two fingers and the thumb) were shaped differently from the other two fingers that were closed.

As with words, a sign can be produced slightly differently across individuals and linguistic contexts (e.g., due to co-articulation)^12,15,16^. Variability was neglected in our vocabulary-based investigation, a limitation not impacting our conclusions concerning biological constraints. Let’s illustrate this point with an example from digit individuation, a type of evidence examined to investigate the biological constraints. The index finger was classified as selected if it was extended while the other fingers and the thumb were flexed. Even if extension and flexion can each vary in this handshape, the index finger must be relatively more extended—an invariable aspect whose violation results in the incorrect articulation of the sign. It was invariable aspects like this that were examined for digit individuation—as well as for the other results concerning the biological constraints.

The hierarchical cluster analysis was conducted on the percentages of occurrence of the 160 distinct handshapes found in each of the 33 languages, using SPSS^®^ software^58^. The selected cluster method was Between Group Linkage; the selected measure option was Squared Euclidean Distances.

## Supplementary Information

Supplementary Information.

## Data Availability

Analyzed data are reported in Supplementary Information A; examples of the handshapes found in the 33 sign languages are included in Supplementary Information B.
