# Supplementary material for: How the hand has shaped sign languages
Source: Sci Rep. 2022 Jul 13;12:11980. doi: 10.1038/s41598-022-15699-1 (PMC9279340; doi:10.1038/s41598-022-15699-1)
Supplement: Supplementary file 1 — Supplementary Information. [file 41598_2022_15699_MOESM1_ESM.pdf]

Supplementary Information A

Supplementary Information A1. List of concepts
List of the 800 concepts for which corresponding signs were searched on online dictionaries.

ABLE, ABOVE, TO ABSORB, TO ACCEPT, ACCIDENT, ACCOUNTING, ADDRESS, TO ADMIRE, TO ADMIT, ADVICE, AFTERNOON, AGAIN, AGAINST, AGE, TO AGREE, AIR, AIRPLANE, TO ALLOW, ALONE, ALWAYS, ANGRY, ANIMAL, TO ANSWER, APPLE, APPOINTMENT, TO ARGUE, TO ARREST, TO ARRIVE, ART, TO ASK, TO ATTACK, ATTENTION, AUNT, AUTUMN, AVAILABLE, TO AVOID, AWAKE, AWFUL, AWKWARD, BABY, BAD, BAG, BALL, BANANA, BANK, BAR, BATHROOM, BATTERY, BEACH, BEAR, BEARD, BEAUTIFUL, BED, BEE, BEER, TO BEHAVE, TO BELIEVE, TO BELONG, BELT, BESIDE, TO BET, BIG, BIKE, BILL, TO BIND, BIRD, BIRTH, TO BITE, BITTER, BLACK, TO BLAME, BLANKET, BLOOD, TO BLOW, BLUE, TO BLUSH, BOAT, BODY, BONE, BOOK, BOOTS, BORED, BOSS, TO BOTHER, BOTTLE, BOWL, BOX, BOY, BRAVE, BREAD, TO BREAK, BREAKFAST, TO BREATHE, BRIDGE, TO BRING, BROOM, BROTHER, BROWN, BUCKET, TO BUILD, BULLY, BUS, BUSY, BUTTERFLY, TO BUY, CAKE, CALM, CAMERA, CAN (NOUN), CAN (VERB), TO CANCEL, CANDLE, CANDY, CAR, TO CARE, CARROT, TO CARRY, CASTLE, CAT, CEILING, CHAIN, CHAIR, CHANCE, TO CHANGE, TO CHASE, CHEAP, TO CHEAT, TO CHECK, CHEESE, CHERRY, CHICKEN, CHOCOLATE, CHOICE, CHURCH, CIGARETTE, CIRCLE, CIRCUS, CITY, CLASS, CLEAN, CLEAR, CLEVER, TO CLIMB, CLOCK, CLOSED, CLOSET, CLOTHES, CLOUD, COFFEE, COLD, COLOR, COMB, TO COME, COMMON, TO COMMUNICATE, TO COMPARE, TO COMPLAIN, COMPLICATED, COMPUTER, CONFUSED, TO CONGRATULATE, TO CONTINUE, TO CONVINCE, TO COOK, TO COOPERATE, COPY, CORN, CORNER, CORRECT, COUCH, TO COUGH, TO COUNT, COUNTRY, COUSIN, TO COVER, COW, CRAZY, TO CREATE, TO CRITICIZE, CROSS, CROWD, TO CRY, CUP, CURIOUS, TO CUT, CUTE, TO DANCE, DANGEROUS, DARK, DAUGHTER, DEAF, DEAR, DEATH, TO DECEIVE, TO DECIDE, DEEP, DELICIOUS, TO DENY, TO DESIRE, TO DESTROY, DIAMOND, DIFFERENT, DIFFICULT, TO DIG, DIRTY, TO DISAGREE, TO DISAPPEAR, DISCOUNT, DISGUST, TO DISTRIBUTE, TO DIVE, TO DIVORCE, DIZZY, DOCTOR, DOG, DOLL, DONKEY, DOOR, TO DREAM, DRESS, TO DRINK, TO DRIVE, TO DROP OUT, DROWN, DRY, DUST, DUTY, EAR, EARLY, TO EARN, EASY, TO EAT, EDUCATION, EFFORT, EGG, ELECTION, ELECTRICITY, ELEGANT, ELEPHANT, ELEVATOR, TO EMBARRASS, EMERGENCY, EMPTY, END, ENEMY, ENERGY, ENGINE, TO ENTER, ENVELOPE, ERROR, TO ESCAPE, TO EVALUATE, EVEN, EVENING, EVIL, TO EXCHANGE, EXCUSE, EXIT, EXPENSIVE, EXPERIENCE, TO EXPLAIN, TO EXPLODE, EYE, FACT, TO FAIL, TO FAINT, FAIR, TO FALL, FAMILY, FAMOUS, FANTASY, FAR, FARM, FAT, FATHER, FAVOR, TO FEED, FENCE, FEVER, TO FIGHT, TO FIND, TO FINISH, FIRE, FISH, FIVE, FLAG, FLAT, FLOOR, FLOWER, TO FLY, TO FOLLOW, FOOD, FOOT, FOREIGN, TO FORGET, TO FORGIVE, FORK, FOX, FREE, FRESH, FRIDGE, FRIEND, FROG, FRUIT, FULL, FUNNY, FURNITURE, FUTURE, GARDEN, GAY, TO GET, GHOST, GIRAFFE, GIRL, TO GIVE, GLASS, GLASSES, GLOVE, GLUE, GOAT, GOD, GOLD, GOOD, GRANDFATHER, GRANDMOTHER, GRAPES, GRASS, GREAT, GREEN, TO GROW, TO GUESS, HABIT, HAIR, HALF, HAMMER, HAND, HAPPY, HARD, HAT, TO HATE, TO HAVE, HEALTHY, HEART, HEAVY, HELICOPTER, TO HELP, TO HIDE, HIGH, TO HIRE, HISTORY, TO HIT, HOLE, HOLIDAY, HONEST, TO HOPE, HORSE, HOSPITAL, HOT, HOTEL, HOUSE, TO HUG, HUMBLE, HUNDRED, HUNGRY, HUSBAND, ICE, ICE-CREAM, IDEA, TO IGNORE, TO IMPORT, IMPORTANT, TO INCREASE, INDEPENDENT, INDUSTRY, INJURY, INNOCENT, INSECT, INSIDE, INSULT, INSURANCE, INTERNATIONAL, TO INVITE, ISLAND, JEALOUS, TO JOIN, JOKE, TO JUMP, JUSTICE, TO KEEP, KEY, TO KILL, KIND, TO KISS, KITCHEN, KNIFE, TO KNOW, LADDER, LAMP, LATE, TO LAUGH, LAW, LAZY, LEAF, TO LEARN, TO LEAVE, LEMON, TO LEND, LENGTH, LETTER, LIBRARY, TO LIE, LIFT, LIGHT, TO LIKE, LION, TO LIVE, LONG, TO LOOK, TO LOVE, LOW, LUCK, LUNCH, MAIN, MAN, MAP, MARKET, MARRIAGE, MASK, MATCH, MATHEMATICS, MEANING, MEAT, TO MEET, MELON, TO MELT, MIDDLE, MILK, MIND, MIRROR, TO MISS, TO MIX, MODERN, MONEY, MONKEY, MONTH, MOON, MORE, MORNING, MOTHER, MOTORCYCLE, MOUNTAIN, MOUSE, MOUTH, MOVEMENT, MOVIE, MUSEUM, MUSHROOM, NAKED, NAME, NAPKIN, NARROW, NASTY, NATION, NATURE, NEAR, NEED, NEGATIVE, NEPHEW, NERVOUS, NEVER, NEW, NEWSPAPER, NICE, NIECE, NIGHT, NORMAL, NOTICE, NOW, NUMBER, TO OBEY, OFFICE, OFTEN, OLD, ONE, ONION, OPEN, OPINION, ORANGE, ORDER, TO ORGANIZE, OUTSIDE, PAGE, PAIN, TO PAINT, PALE, PAN, PANTS, PARENTS, PARK (NOUN), TO PARK, PARROT, TO PARTICIPATE, PARTY, PASSPORT, PAST, PATH, TO PAY, PEACE,

PEAR, PEN, PENGUIN, PEOPLE, PERCENTAGE, PERFECT, PERFORM, PERSON, TO PICK, PICTURE, PIG, PILLOW, PINK, PIZZA, PLANT, PLASTIC, PLATE, TO PLAY, PLEASURE, PLUG, POLICE, POLITE, POLITICS, POLLUTION, POOR, POSITIVE, POTATO, TO POUR, TO PREFER, TO PREPARE, PRESENT, PRESSURE, TO PRETEND, PRICE, TO PRINT, PRISON, PRIVATE, PRIZE, PROBLEM, PROMISE, TO PROTECT, TO PROTEST, PROUD, TO PUNISH, PURPLE, TO PUSH, TO PUT, QUALITY, QUICK, QUIET, RABBIT, RACE, RAIN, RAINBOW, TO REACH, TO READ, READY, REASON, TO RECOGNIZE, RED, TO REDUCE, TO RELAX, TO RELEASE, TO REMEMBER, RENT, TO REPAIR, TO REPEAT, TO RESIGN, TO RESPECT, RESTAURANT, RESULT, TO RETURN, RICE, RICH, RISK, RIVER, ROAD, ROCKET, ROLL, ROOF, ROOM, ROPE, ROUGH, TO RUB, RUDE, RULER, TO RUN, SAD, SAFE, SALAD, SALARY, SALT, SALUTE, SAME, SANDWICH, TO SAVE, SCARED, SCARF, SCHEDULE, SCHOOL, SCIENCE, SCISSORS, TO SCOLD, SCREWDRIVER, SEA, TO SEARCH, SECRET, SELFISH, TO SELL, TO SEND, TO SEPARATE, SERIOUS, TO SEW, TO SHAKE, TO SHARE, SHARP, TO SHAVE, SHEEP, SHINY, SHIRT, SHOCK, SHOES, TO SHOOT, SHORT, TO SHOW, TO SHUT UP, SHY, SICK, SIGN LANGUAGE, SIGNATURE, SILVER, SIMPLE, TO SING, SISTER, TO SIT, SIZE, TO SKATE, TO SKI, SKIN, SKIRT, TO SLIP, SLIPPERY, SLOW, SMALL, SMELL, SMOOTH, SNAKE, SNOW, SOAP, SOCCER, SOCKS, SOFT, SOLDIER, SON, SOON, SORRY, SOUP, TO SPEAK, SPECIAL, SPEED, TO SPEND, SPICY, SPIDER, SPOON, SPORT, SPRAY, SPRING, SQUARE, SQUIRREL STAR, TO START, STATION, TO STAY, TO STEAL, TO STINK, STONE, STOP, STORE, STORM, STRAIGHT, STRANGE, STRICT, STRONG, STUBBORN, STUDENT, TO STUDY, STUPID, TO SUFFER, SUITCASE, SUMMER, SUN, SUPPORT, SURPRISE, SUSPECT, SWALLOW, TO SWEAT, SWEET, TO SWIM, SWING, TABLE, TAIL, TO TAKE, TALL, TASTE, TAX, TEA, TO TEACH, TEAM, TELEPHONE, TEN, TENT, THANK, THEATER, THERMOMETER, THICK, THIN, TO THINK, THIRSTY, THREATEN, THREE, TICKET, TIGER, TIGHT, TIME, TIRED, TODAY, TOGETHER, TOMATO, TOMORROW, TONGUE, TOOTHBRUSH, TOPIC, TO TOUCH, TOWEL, TOWN, TOY, TRACTOR, TRAFFIC, TRAIN, TRAINING, TO TRAVEL, TREE, TRUCK, TO TRUST, TRUTH, TRY, TO TURN, TURTLE, TWINS, TWO, UGLY, UMBRELLA, UNCLE, UNDER, TO UNDERSTAND, UNIVERSITY, TO USE, VEGETABLE, TO VISIT, TO WAIT, WALK, WALL, TO WANT, WAR, TO WASH, WATER, WAVE, WEAK, WEATHER, WEEK, WEIGHT, WET, WHITE, WIFE, TO WIN, WIND, WINDOW, WINE, WINTER, WITHOUT, WOLF, WOMAN, WOOD, TO WORK, WORM, TO WORRY, TO WRITE, WRONG, YEAR, YELLOW, YESTERDAY, YOUNG.

Supplementary Information A2. Sources consulted for signs

Websites of the online dictionaries listing the signs in each of the 33 sign languages that we analyzed. Lexical searched were conducted using written English translations, with the exception of a few sign languages (Danish, Norwegian, Mexican) for which native speakers were consulted for translations in other written languages than English.

Spread the Sign (www.spreadthesign.com)

American, Argentinian, Austrian, Belarusian, British, Chinese, Croatian, Czech, Estonian, French, German, Icelandic, Indian, Italian, Japanese, Latvian, Lithuanian, Mexican, Polish, Portuguese, Romanian, Russian, Slovak, Spanish, Swedish, Turkish, Ukrainian, Urdu

Auslan Signbank (www.auslan.org.au/)

Auslan

New Zealand Sign Language Dictionary (www.nzsl.nz/)

New Zealand sign language

Ordbog over Dansk Tegnsprog (www.tegnsprog.dk/)

Danish

Tegnordbok.no (www.minetegn.no/Tegnordbok-2016/)

Norwegian

TSL Online Dictionary (http://140.123.46.77/TSL/indexEN.html)

Taiwanese

Wikisigns (www.wikisigns.org/statistics)

Mexican

Indian Sign Language Portal (https://indiansignlanguage.org)

Indian

PSL Dictionary (www.psl.org.pk/signs)
Urdu

Supplementary Information A3. Sign corpus

The following information for the signs in 33 languages is reported in the table below:

a. *Concepts with Signs (%)*, percentage of concept (n=800) for which a corresponding sign was found
b. *Scored Signs (N)*, number of analyzed signs
c. *Excluded Signs (N)*, number of signs excluded from the analyses because a word was fingerspelled, a sentence was used to describe the concept, or the sign was not fully visible on the video
d. *Handshapes (N)*, number of handshapes analyzed for the dominant hand and the non-dominant hand, respectively

Sign Language (SL)	Concepts with Signs (%)	Scored Signs (N)	Excluded Signs (N)	Handshapes (N)/Hand	
				Dominant	Non-Dominant
American	99.4	834	12	1,054	284
Argentinian	96.8	843	2	1,278	213
Australian	97.9	1,192	5	1,458	361
Austrian	99.1	824	1	995	188
Belarusian	99.9	857	11	1,468	267
British	99.9	806	20	1,047	255
Chinese	98.4	817	2	1,320	265
Croatian	99.3	819	0	1,027	207
Czech	99.4	801	3	985	210
Danish	96.3	798	0	933	161
Estonian	99.9	801	0	1,027	238
French	98.5	787	4	1,052	247
German	100.0	874	0	1,047	173
Icelandic	99.8	795	4	1,066	232
Indian	91.3	844	26	1,505	274
Italian	99.6	931	5	1,306	236
Japanese	94.6	806	2	988	243
Latvian	99.4	848	1	1,087	218
Lithuanian	99.5	840	6	1,132	199
Mexican	87.3	729	2	955	256
New Zealand SL	99.5	1,090	2	1,325	243
Norwegian	96.4	1,013	0	1,191	223
Polish	99.8	877	7	1,158	275
Portuguese	99.4	823	2	1,212	224
Romanian	90.9	677	65	870	161
Russian	99.6	827	5	1,082	246
Slovak	97.8	822	0	1,021	174
Spanish	99.6	815	1	1,112	191
Swedish	99.9	943	5	1,180	262
Taiwanese	86.8	888	7	1,195	317
Turkish	99.0	1,023	4	1,426	276
Ukrainian	99.5	869	6	1,189	245
Urdu	98.9	833	11	1,344	204
Total	–	28,343	221	38,035	7,768

Supplementary Information A4. Independence scores

An independence score was calculated from the signs in 33 languages. The independence score of the thumb corresponded to the percentage of handshapes in which the thumb was shaped differently compared to the fingers. The independence score of a finger corresponded to the percentage of handshapes in which the finger was shaped differently compared to the thumb and the other three fingers.

Sign Language (SL)	Independence Score/Digit				
	Thumb	Index Finger	Middle Finger	Ring Finger	Little Finger
American	31.19	13.18	1.61	0.00	0.85
Argentinian	26.92	11.42	0.47	0.00	0.70
Australian	33.22	15.02	0.68	0.00	1.51
Austrian	26.55	10.68	1.73	0.00	0.30
Belarusian	28.12	14.10	0.54	0.00	0.14
British	29.05	14.05	0.38	0.00	2.01
Chinese	27.55	14.38	0.23	0.00	1.74
Croatian	27.26	10.22	1.36	0.00	0.10
Czech	27.29	10.55	1.52	0.00	0.30
Danish	31.60	13.07	0.22	0.00	0.64
Estonian	27.46	14.03	0.68	0.00	0.39
French	27.81	10.17	1.34	0.00	0.29
German	27.33	9.56	0.48	0.00	0.67
Icelandic	33.57	11.54	0.28	0.00	0.38
Indian	26.78	13.89	0.80	0.00	0.66
Italian	27.13	9.73	1.30	0.00	0.92
Japanese	25.99	11.94	0.20	0.00	2.02
Latvian	30.15	12.98	0.92	0.00	0.64
Lithuanian	25.66	12.27	1.41	0.00	0.97
Mexican	25.11	11.60	1.05	0.00	2.30
New Zealand SL	34.28	13.21	0.68	0.00	1.43
Norwegian	34.57	15.12	0.59	0.00	0.08
Polish	27.55	15.72	0.78	0.00	0.43
Portuguese	28.78	13.86	1.15	0.00	0.99
Romanian	29.27	13.20	0.34	0.00	1.26
Russian	24.76	13.30	1.48	0.00	0.37
Slovak	29.39	10.87	0.98	0.00	0.20
Spanish	23.39	10.61	0.99	0.00	1.53
Swedish	35.06	13.39	0.17	0.00	0.42
Taiwanese	36.65	12.55	1.09	0.84	1.67
Turkish	27.96	10.93	0.77	0.00	0.91
Ukrainian	21.95	13.21	1.51	0.00	0.17
Urdu	28.11	14.14	0.22	0.00	0.67
Average	28.71	12.56	0.85	0.03	0.84

Supplementary Information A5. Neighboring vs. distant fingers

We examined two types of finger pairs:

a. Adjacent Finger Pairs included immediately neighboring fingers (index and middle fingers, middle and ring fingers, ring and little fingers)
b. Non-adjacent Finger Pairs included distant fingers (index and ring fingers, index and little fingers, middle and little fingers)

The next two tables report the percentages, in 33 languages, of handshapes in which the fingers in the pair were selected (i.e., shaped differently compared to the other two fingers and the thumb that were closed; Table A), or identically shaped (Table B).

Table A. Pairs with selected fingers

Sign Language (SL)	% Handshapes/Two Fingers	
	Adjacent	Non-adjacent
American	3.35	0.09
Argentinian	1.78	0.24
Australian	2.31	0.09
Austrian	3.66	0.00
Belarusian	2.57	0.32
British	2.52	0.13
Chinese	2.50	0.08
Croatian	3.09	0.03
Czech	3.92	0.00
Danish	2.50	0.00
Estonian	2.17	0.42
French	3.05	0.13
German	3.15	0.03
Icelandic	2.44	0.00
Indian	2.30	0.07
Italian	2.45	0.23
Japanese	2.49	0.20
Latvian	2.63	0.37
Lithuanian	2.98	0.62
Mexican	4.88	0.24
New Zealand SL	2.09	0.08
Norwegian	2.18	0.03
Polish	2.89	0.06
Portuguese	2.70	0.06
Romanian	3.36	0.00
Russian	2.70	0.61
Slovak	3.07	0.03
Spanish	2.61	0.27
Swedish	2.76	0.03
Taiwanese	2.25	0.36
Turkish	3.34	0.16
Ukrainian	3.28	0.56
Urdu	2.70	0.00
Average	2.81	0.17

Table B. Pairs with identically shaped fingers

Sign Language (SL)	% Handshapes/Two Fingers	
	Adjacent	Non-adjacent
American	45.06	37.10
Argentinian	37.80	31.51
Australian	47.75	39.32
Austrian	38.55	31.36
Belarusian	41.48	33.57
British	42.94	34.82
Chinese	41.46	33.25
Croatian	38.39	31.64
Czech	39.35	31.92
Danish	44.00	36.53
Estonian	40.74	33.14
French	39.84	32.32
German	38.01	31.16
Icelandic	44.35	37.79
Indian	40.17	32.27
Italian	39.01	31.78
Japanese	38.36	31.08
Latvian	43.15	35.67
Lithuanian	39.03	31.63
Mexican	41.03	30.72
New Zealand SL	46.60	39.72
Norwegian	47.47	40.06
Polish	42.01	33.51
Portuguese	42.70	34.55
Romanian	43.53	34.35
Russian	38.70	30.92
Slovak	40.99	33.77
Spanish	35.29	28.37
Swedish	47.42	39.80
Taiwanese	49.88	43.12
Turkish	40.62	32.58
Ukrainian	36.21	27.98
Urdu	41.47	33.22
Average	41.62	33.96

Supplementary Information A6. Grasp-like handshapes

Out of the 160 distinct handshapes observed in 33 sign languages, we identified 13 handshapes in which the thumb and the fingers had the same configuration as in 12 of the grasps included in the GRASP Taxonomy of Feix et al. (1). The grasp-like handshapes are illustrated below, and corresponded to different types of grasps – power grasps, intermediate grasps, and precision grasps. For each grasp-like handshape, we report the number used in the GRASP Taxonomy of Feix et al.[1] to identify the grasp. Pictures are from the website *Spread the Sign*[2].

Power Grasps

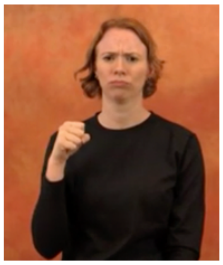
3

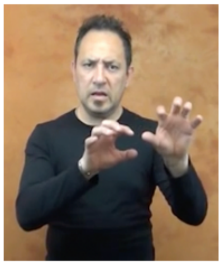
11

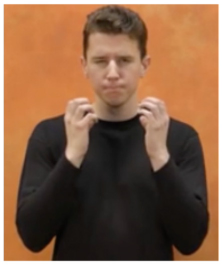
10

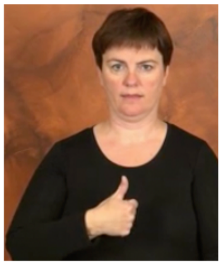
5

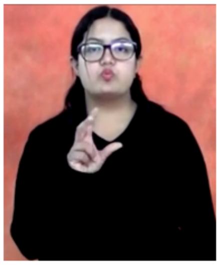
28

Intermediate Grasps

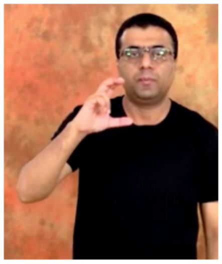
23

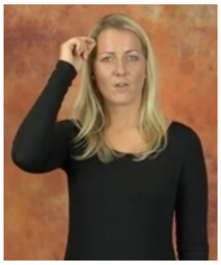
16

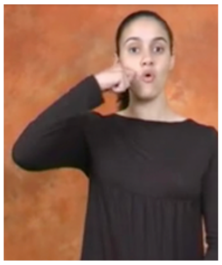
32

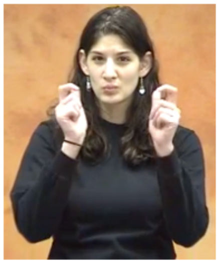
25

Precision Grasps

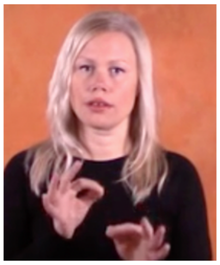
9

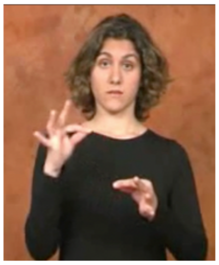
9

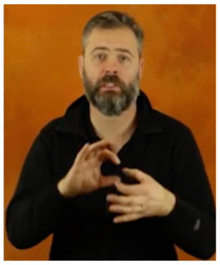
33

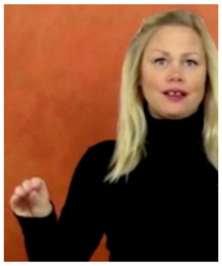
22

Supplementary Information A7. Handshapes corresponding to power or precision grasps
Percentage of handshapes in which the thumb and the fingers had the same configuration as in the power grasps or the precision grasps. The distinction between power and precision grasps is based on the GRASP Taxonomy of Feix et al.[1] The grasp-like handshapes are illustrated in Supplementary Information A7.

Sign Language (SL)	% Grasp-like Handshapes/Type of Grasp	
	Power	Precision
American	15.09	3.42
Argentinian	12.13	5.71
Australian	14.81	3.36
Austrian	13.94	4.58
Belarusian	9.74	5.79
British	13.37	3.44
Chinese	10.67	3.18
Croatian	14.02	4.38
Czech	12.39	5.08
Danish	13.93	3.86
Estonian	15.09	2.92
French	12.74	4.09
German	14.71	5.25
Icelandic	15.95	3.19
Indian	12.96	5.71
Italian	13.40	3.45
Japanese	15.08	4.76
Latvian	13.06	3.59
Lithuanian	13.60	4.86
Mexican	11.52	2.83
New Zealand SL	14.94	4.15
Norwegian	16.04	3.78
Polish	14.25	5.79
Portuguese	13.28	3.71
Romanian	14.02	4.83
Russian	10.63	4.16
Slovak	12.05	6.17
Spanish	12.32	3.78
Swedish	13.22	2.03
Taiwanese	15.31	5.19
Turkish	13.18	6.45
Ukrainian	11.69	4.54
Urdu	13.39	5.21
Average	13.41	4.34

Supplementary Information A8. Sources consulted for manual alphabets

The websearch for the manual alphabets associated with sign languages was conducted using the search terms “sign language,” “fingerspelling,” and “manual alphabet” in the corresponding written language (e.g., in Russian for the Russian manual alphabet). Whenever a translation was needed, we used *Goggle Translator*. We only analyzed letter handshapes from websites in which the letter signs were presented along with the corresponding written letter. Whenever possible, we consulted two websites for each manual alphabet.

American	https://www.youtube.com/watch?v=tkMg8g8vVUo
	https://www.youtube.com/watch?v=cGavOVNDj1s
Arabic	https://www.youtube.com/watch?v=6w17Pa6TCG8&t=760s
	https://www.youtube.com/watch?v=fJry64Z-6CA
Argentinian	https://www.youtube.com/watch?v=FZxmGh9Ij7g
	https://www.youtube.com/watch?v=Iqbghn2eXGM
Australian	https://www.auslan.org.au/
	https://www.youtube.com/watch?v=AFP4a3foXLA
Austrian	https://www.youtube.com/watch?v=CxnKgOiNQ38
	https://www.youtube.com/watch?v=4T9TGZSaNcM
Bolivian	https://www.youtube.com/watch?v=zjswxA0GsIw
	https://www.youtube.com/watch?v=7xTg0HxwIcw
Brazilian	https://www.youtube.com/watch?v=fYaXJXf60gU&t=4s
	https://www.youtube.com/watch?v=wwK_QHRmz8A
British	https://www.youtube.com/watch?v=mkTeqA4kwUQ
	https://www.youtube.com/watch?v=C_dbN9N0pR4
Bulgarian	https://www.youtube.com/watch?v=8YmTxIp2Gtg
	https://www.spreadthesign.com/bg.bg/alphabet/20/
Chilean	https://www.youtube.com/watch?v=gvEbKlH2g4o
	https://www.youtube.com/watch?v=qMx_y4ZYYbg
Colombian	https://www.youtube.com/watch?v=JMraBJsA9oI&t=134s
	https://www.youtube.com/watch?v=IAG3OxPOUp0
Costa Rican	https://www.youtube.com/watch?v=fhNi4L5xScI&t=60s
	https://www.youtube.com/watch?v=Vw069dT12wU
Croatian	https://www.youtube.com/watch?v=lYoKQRJygyY&t=9s
	https://www.youtube.com/watch?v=B42SDLVWaz8&t=4s
Cuban	https://www.youtube.com/watch?v=-K0Eu9Sx2OA&t=150s
Czech	https://www.youtube.com/watch?v=IUsCjVfBuFc
	https://www.youtube.com/watch?v=hFuf5L15u2o
Danish	https://www.youtube.com/watch?v=--zEog24FS0
Dominican	https://www.youtube.com/watch?v=nQ_lRsh8n3U
	https://www.youtube.com/watch?v=h7RGrub4q34
Dutch	https://www.youtube.com/watch?v=6x7YGMznZaM
	https://www.youtube.com/watch?v=6x7YGMznZaM
Ecuadorian	https://www.youtube.com/watch?v=qMxNSNJF3Oc
	https://www.youtube.com/watch?v=K6tuWefifxc
Estonian	https://www.youtube.com/watch?v=TrtZlJqhgq4
	https://www.spreadthesign.com/et.ee/alphabet/19/
Filipino	https://www.youtube.com/watch?v=iYpTJ5cEl9Y&t=12s
	https://www.youtube.com/watch?v=jVLJ63NkRPs
Finnish	https://www.youtube.com/watch?v=g_tKA-uxoAo
	https://www.youtube.com/watch?v=5adBxNMsgJ0
French	https://www.youtube.com/watch?v=jg5zXcN2tlY
	https://www.youtube.com/watch?v=cgae3B7n-co
German	https://www.youtube.com/watch?v=6Swqlj7eNNQ
	https://www.youtube.com/watch?v=Cn2Nle9Vca0
Greek	https://www.youtube.com/watch?v=atPVTWAprlQ
	https://www.youtube.com/watch?v=cow87wA1RhY
Guatemalan	https://www.youtube.com/watch?v=d1MA68BvTZo

	https://www.youtube.com/watch?v=uX7QQA7QYvQ
Hebrew	https://www.youtube.com/watch?v=Y_9sY1P6UxM&t=30s
	https://www.youtube.com/watch?v=29aK86-6b8I
Hungarian	https://www.youtube.com/watch?v=eFY8KuBQIrM
	https://www.youtube.com/watch?v=-Tzc1e-LsRw
Honduran	https://www.youtube.com/watch?v=dDtR0MeGi5o
	https://www.youtube.com/watch?v=0gUVgNzvzQw
Icelandic	https://www.youtube.com/watch?v=NPzooxWrp0o
	https://www.youtube.com/watch?v=SwyfWCQiGyA
Indian	https://www.youtube.com/watch?v=WrjfUvmWzko
	https://www.youtube.com/watch?v=Vj_13bdU4dU
Indonesian	https://www.youtube.com/watch?v=pjaBEouX_8g
	https://www.youtube.com/watch?v=F0WrOSmry6g
Irish	https://www.youtube.com/watch?v=Jgjn_hbKiYU
	https://www.youtube.com/watch?v=pNdlrKrWWPo
Italian	https://www.youtube.com/watch?v=0Yx9IkOxFyI
	https://www.youtube.com/watch?v=Czi28nWReFA
Japanese	https://www.youtube.com/watch?v=MhV95_n1Qcs&t=38s
	https://www.youtube.com/watch?v=zuwc6pfHtGk
Korean	https://www.youtube.com/watch?v=Zl9pmyZ31p8
	https://www.youtube.com/watch?v=1lbg4Kck_AI
Latvian	https://www.youtube.com/watch?v=V7zSIDPi_Rs
	https://www.spreadthesign.com/lv.lv/alphabet/27/
Lithuanian	https://www.youtube.com/watch?v=NvuF6xX-fQQ
	https://www.youtube.com/watch?v=CXu7tN2etAE
Malaysian	https://www.youtube.com/watch?v=EvPNapJzpdM
	https://www.youtube.com/watch?v=zUxKigxzgkk
Mexican	https://www.youtube.com/watch?v=mF1-52pq9sQ
	https://www.youtube.com/watch?v=pn_gJfVibx4
New Zealand SL	https://www.youtube.com/watch?v=xiRqC-aM5xM
	https://www.youtube.com/watch?v=SRuq8TH-MyI
Nicaraguan	https://www.youtube.com/watch?v=J7q73qeatlw
	https://www.youtube.com/watch?v=QOEfE2CIR3E
Norwegian	https://www.youtube.com/watch?v=xVFLfKcRAyc
	https://www.youtube.com/watch?v=wjjTGvZBLi8&t=265s
Panamanian	https://www.youtube.com/watch?v=2bCvtWSuzyU
	https://www.youtube.com/watch?v=_3MkbS1hddc
Paraguayan	https://www.youtube.com/watch?v=8KUz7d0h7M8
	https://www.youtube.com/watch?v=Cg0SQpYS11M
Peruvian	https://www.youtube.com/watch?v=xEsI4vFBLSQ&t=77s
	https://www.youtube.com/watch?v=fXPnB05SXUo
Polish	https://www.youtube.com/watch?v=0KqQZyrPYQg
	https://www.youtube.com/watch?v=nTHYuPaSMRo
Portuguese	https://www.youtube.com/watch?v=OVqH10VsIgg
	https://www.youtube.com/watch?v=02DrxsYsFI0
Romanian	https://www.youtube.com/watch?v=t1vdkZqQX5U
	https://www.youtube.com/watch?v=G_dkpklT1H0&list=PL3533A40DF32F54D6&index=3
Russian	https://www.youtube.com/watch?v=OZjiYcpD-5w&t=143s
	https://www.youtube.com/watch?v=jtbwEalS0CE
Salvadoran	https://www.youtube.com/watch?v=Y6e1n6s19aM
	https://www.youtube.com/watch?v=3PkHTpFhjXg
Singaporean	https://www.youtube.com/watch?v=Xx3fgCP_5sk
	https://www.youtube.com/watch?v=6EW4PVT_d_4
Slovakian	https://www.youtube.com/watch?v=8SwyGYbsXu4
	https://www.youtube.com/watch?v=rPboM8_cYo0
Slovenian	https://www.youtube.com/watch?v=IOzB7ssoO7s

	https://www.youtube.com/watch?v=qRmD_RIgVAY
South African	https://www.youtube.com/watch?v=D9AuGFiZqCo
	https://www.youtube.com/watch?v=wkUn9wAd4EQ
Spanish	https://www.youtube.com/watch?v=BfPBj4QrtwY
	https://www.youtube.com/watch?v=RRXivEGosk4
Swedish	https://www.youtube.com/watch?v=0TGvDI9hoPk
	https://www.youtube.com/watch?v=_-oH5UesTSU&t=29s
Turkish	https://www.youtube.com/watch?v=f-Q1LelSbVA
	https://www.youtube.com/watch?v=zWJg9Sc6OAs
Ukrainian	https://www.youtube.com/watch?v=DHo96OMHRyE
	https://www.spreadthesign.com/uk.ua/alphabet/15/
Urdu	https://www.youtube.com/watch?v=xbaX0BIHQD4
	https://www.youtube.com/watch?v=cmG4wL7eGjQ
Uruguayan	https://www.youtube.com/watch?v=JUdPvn3LyzA
	https://www.youtube.com/watch?v=OCFYPVixwh8
Venezuela	https://www.youtube.com/watch?v=w5ORV5UZj4A
	https://www.youtube.com/watch?v=UPaYIyLip_Q&t=465s
Vietnamese	https://www.youtube.com/watch?v=qbunwMeiwbg&t=18s
	https://www.youtube.com/watch?v=9nCAAUqAN24

Supplementary Information A10. Handshapes
For each of the 160 distinct handshapes found in the 33 that we analyzed languages, we report the percentage of occurrence and the coefficient of variation calculated across the 33 languages. Handshapes are ordered according to their cross-linguistic frequencies – from the most frequent to the least frequent. The handshapes are shown in SI B.

Frequency Ranking	%	Coefficient of Variation
1	9.926	0.21
2	9.445	0.19
3	8.523	0.13
4	6.841	0.24
5	4.569	0.22
6	3.718	0.28
7	3.497	0.43
8	2.910	0.62
9	2.853	0.21
10	2.710	0.33
11	2.704	0.30
12	2.667	0.46
13	2.550	0.38
14	2.316	0.39
15	1.876	0.27
16	1.686	0.33
17	1.669	0.41
18	1.669	0.31
19	1.480	0.32
20	1.387	0.41
21	1.387	0.67
22	1.304	0.69
23	1.298	0.39
24	1.230	0.40
25	1.182	0.36
26	1.094	0.54
27	1.054	0.50
28	1.002	0.35
29	0.877	0.62
30	0.800	0.73
31	0.789	0.63
32	0.757	1.20
33	0.721	0.45
34	0.576	0.77
35	0.566	1.00
36	0.552	0.62
37	0.538	0.56
38	0.508	0.85
39	0.507	2.00
40	0.430	1.24
41	0.429	1.95
42	0.356	0.78
43	0.343	0.89
44	0.334	0.78

45	0.298	0.91
46	0.284	0.65
47	0.267	0.75
48	0.257	1.20
49	0.254	1.06
50	0.232	1.07
51	0.232	0.81
52	0.230	0.84
53	0.227	0.86
54	0.208	1.02
55	0.197	1.45
56	0.188	0.78
57	0.147	0.86
58	0.146	0.99
59	0.137	1.30
60	0.128	0.92
61	0.125	1.34
62	0.122	1.44
63	0.117	1.42
64	0.108	1.44
65	0.103	1.09
66	0.101	1.10
67	0.095	3.42
68	0.091	1.00
69	0.087	2.00
70	0.084	1.00
71	0.081	5.05
72	0.080	1.39
73	0.075	1.40
74	0.073	1.52
75	0.071	1.00
76	0.065	1.44
77	0.064	1.80
78	0.059	1.31
79	0.050	1.76
80	0.049	4.24
81	0.048	1.59
82	0.043	1.39
83	0.043	2.99
84	0.042	1.92
85	0.040	1.53
86	0.040	2.56
87	0.039	5.43
88	0.037	1.99
89	0.035	2.66
90	0.032	2.22
91	0.032	3.23
92	0.030	2.98
93	0.029	2.07

94	0.029	2.56
95	0.027	5.09
96	0.026	5.74
97	0.025	1.56
98	0.025	3.04
99	0.023	2.12
100	0.022	1.87
101	0.022	3.57
102	0.020	3.00
103	0.019	2.73
104	0.018	5.74
105	0.018	4.81
106	0.017	2.56
107	0.017	3.28
108	0.017	3.28
109	0.017	2.63
110	0.017	2.16
111	0.017	2.18
112	0.016	3.82
113	0.015	3.18
114	0.014	2.44
115	0.014	4.87
116	0.014	5.74
117	0.013	3.50
118	0.011	4.00
119	0.011	3.25
120	0.010	2.75
121	0.009	5.74
122	0.009	5.74
123	0.009	4.49
124	0.008	3.24
125	0.008	4.49
126	0.008	3.27
127	0.008	3.24
128	0.007	5.74
129	0.006	5.74
130	0.006	5.74
131	0.006	5.74
132	0.006	4.01
133	0.006	4.00
134	0.006	4.00
135	0.006	4.01
136	0.005	4.00
137	0.005	4.01
138	0.005	5.74
139	0.003	5.74
140	0.003	5.74
141	0.003	5.74
142	0.003	5.74

143	0.003	5.74
144	0.003	5.74
145	0.003	5.74
146	0.003	5.74
147	0.003	5.74
148	0.003	5.74
149	0.003	5.74
150	0.003	5.74
151	0.003	5.74
152	0.003	5.74
153	0.003	5.74
154	0.003	5.74
155	0.003	5.74
156	0.002	5.74
157	0.002	5.74
158	0.002	5.74
159	0.002	5.74
160	0.002	5.74

References

1. Feix, T., Romero, J., Schmiedmayer, H. B., Dollar, A. M., & Kragic, D. (2015). The grasp taxonomy of human grasp types. *IEEE Transactions on human-machine systems*, *46*(1), 66-77.
2. *Spread the Sign*. https://www.spreadthesign.com

Supplementary Information B

The pictures included in Supplementary Material B illustrate the 160 distinct handshapes identified in the 28,343 signs examined in 33 languages. Each picture shows a screenshot taken from a video available on one of the websites listed in Supplementary Material A2. The screenshots were edited to enhance the presentation of the handshape. While the handshapes were clearly visible on the videos, details may not be discernable in the pictures – missing information is supplied by the descriptions accompanying the pictures. In pictures in which the two hands make different handshapes, we intend to illustrate the handshape of the right hand. We used the terminology of the Hamburg Notation System, after which our analysis was modelled, with the exception of thumb position that was classified using the terms from biomechanics and illustrated in Figure 1. A further terminological note: *digits* refers to the thumb and the four fingers.

The presentation of the handshapes is organized according to the number of selected digits and the shape of the fingers and the thumb, features that were analyzed in the study. The handshapes are grouped in categories within which they share common features. Complex handshapes requiring detailed descriptions are presented separately. Percentages refer to the occurrences of the handshapes in the corpus of 28,343 signs.

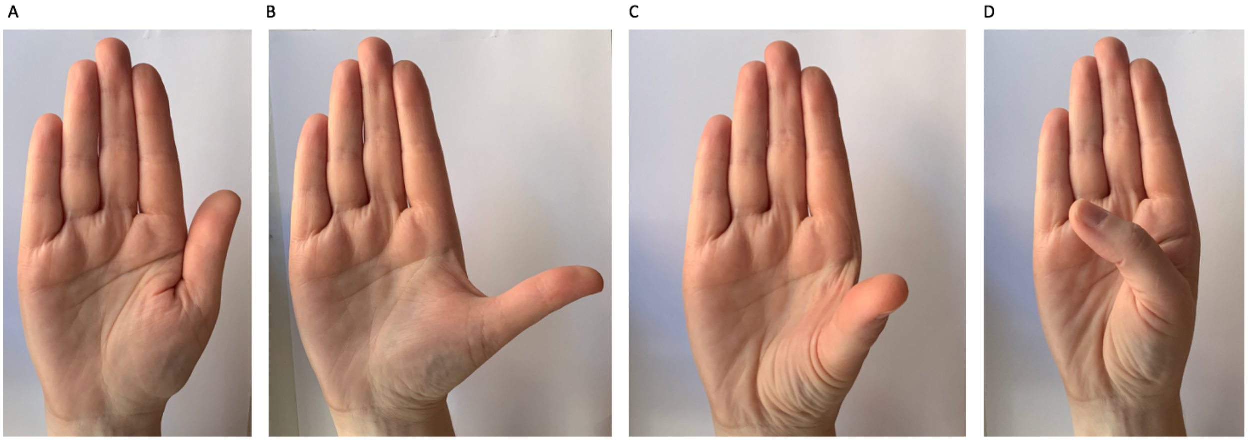

Fig1. Thumb positions: adduction (A), extension (B), abduction (C), and flexion (D).

1 All fingers selected

Fingers are identically shaped. A primary distinction among the handshapes in which all fingers are selected and identically shaped concerns whether or not the fingers make contact with the thumb.

1.1a Thumb-finger contact

The thumb makes contact with multiple fingers or (primarily) with the index finger. Finger shape varies – fingers are all closed, bent, flattened, or stacked.

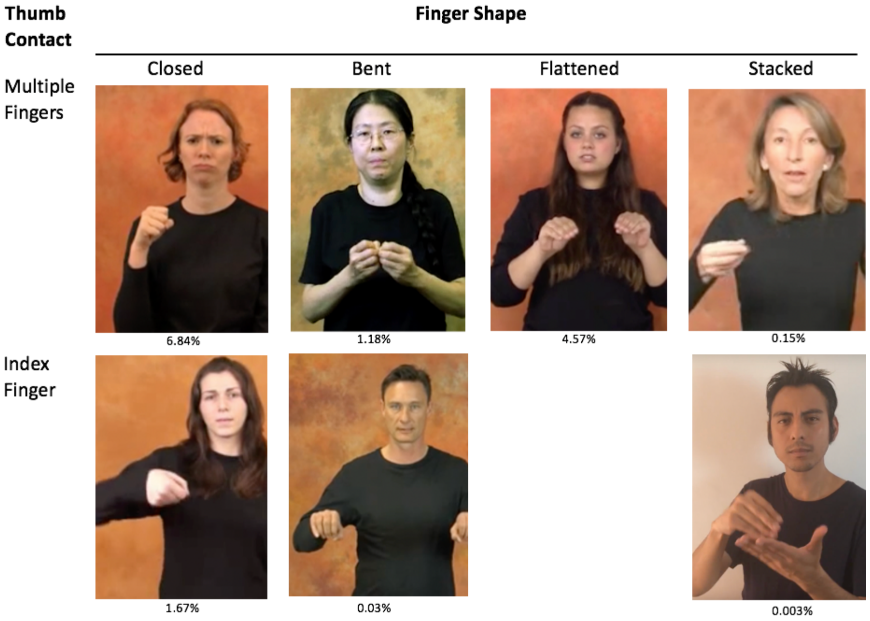

1.1b Complex handshapes

In A, the thumb is in between the fingers. In B, the fingers are stacked and the thumb is extended.

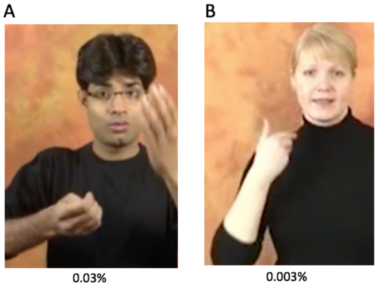

1.2 No thumb-finger contact

Fingers are all selected and identically shaped and do not make contact with the thumb. They vary depending on whether or not they are spread. Their shape also varies – they are extended, bent, flattened, or hooked. The thumb is extended, abducted, adducted, or flexed.

1.2.a Spread fingers

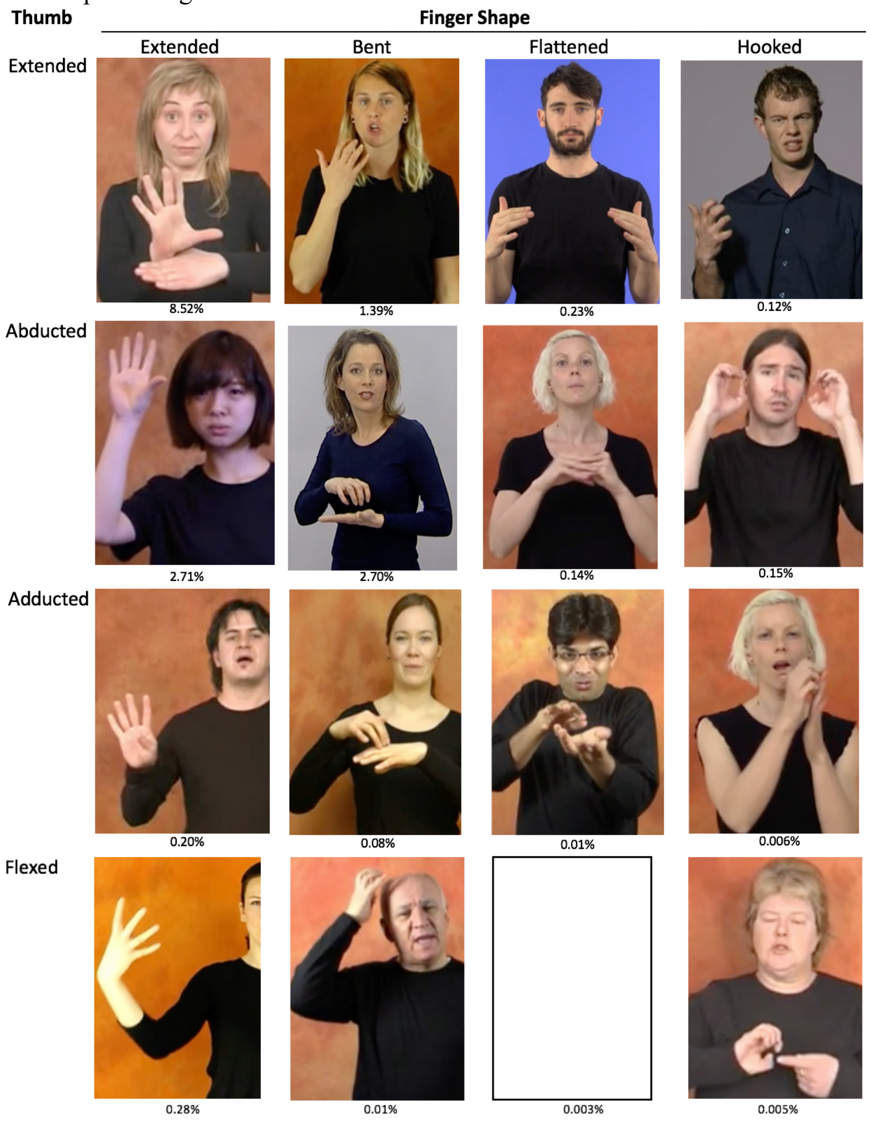

Note: The picture of the handshape with flattened fingers and flexed thumb is missing.

1.2b Non-spread fingers

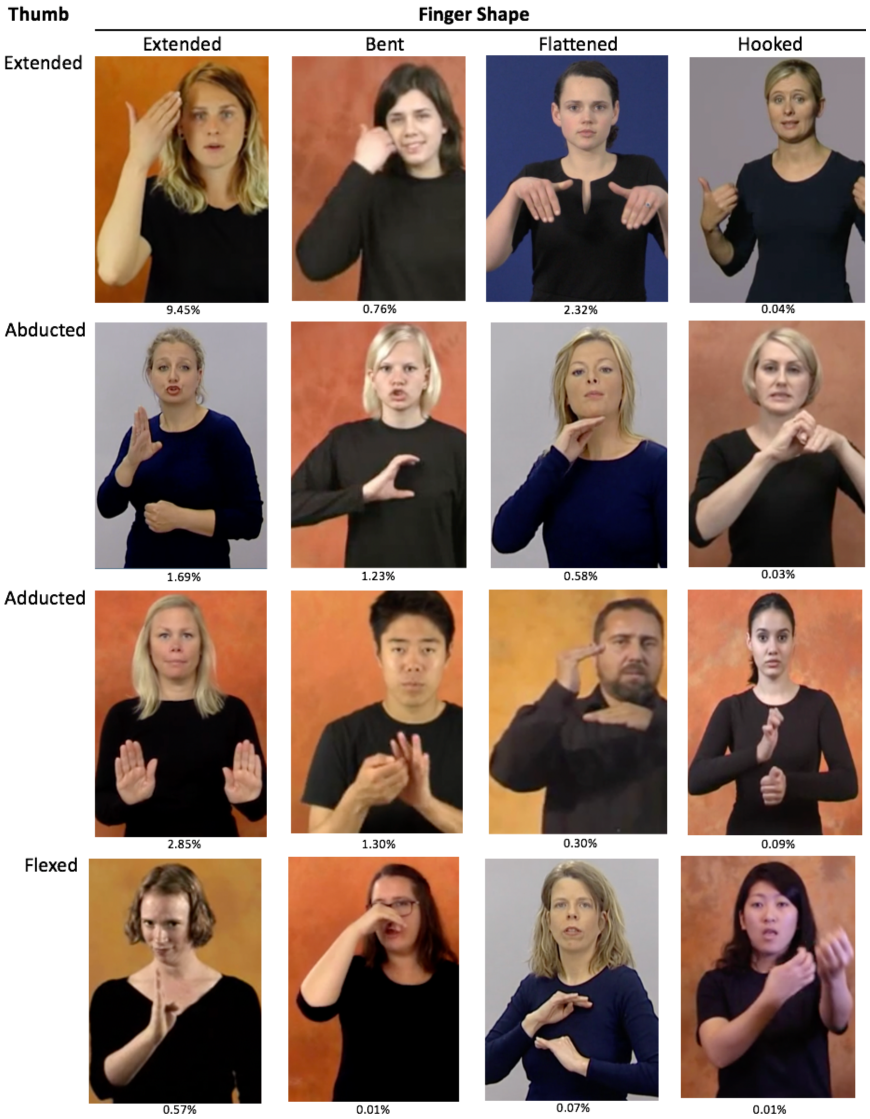
Thumb
Finger Shape
Extended
Bent
Flattened
Hooked
Extended
9.45%
0.76%
2.32%
0.04%
Abducted
1.69%
1.23%
0.58%
0.03%
Adducted
2.85%
1.30%
0.30%
0.09%
Flexed
0.57%
0.01%
0.07%
0.01%

2. One selected finger or selected thumb

The selected finger or the selected thumb is shaped differently compared to the other digits. Shapes of the selected and non-selected digits vary.

2.1 Closed non-selected digits

One finger or the thumb is shaped differently compared to the other digits that are closed. The thumb is either extended or bent. Each of the fingers can be selected in this type of handshapes. All selected fingers can be extended; only the index and the little fingers are shaped in additional configurations when selected.

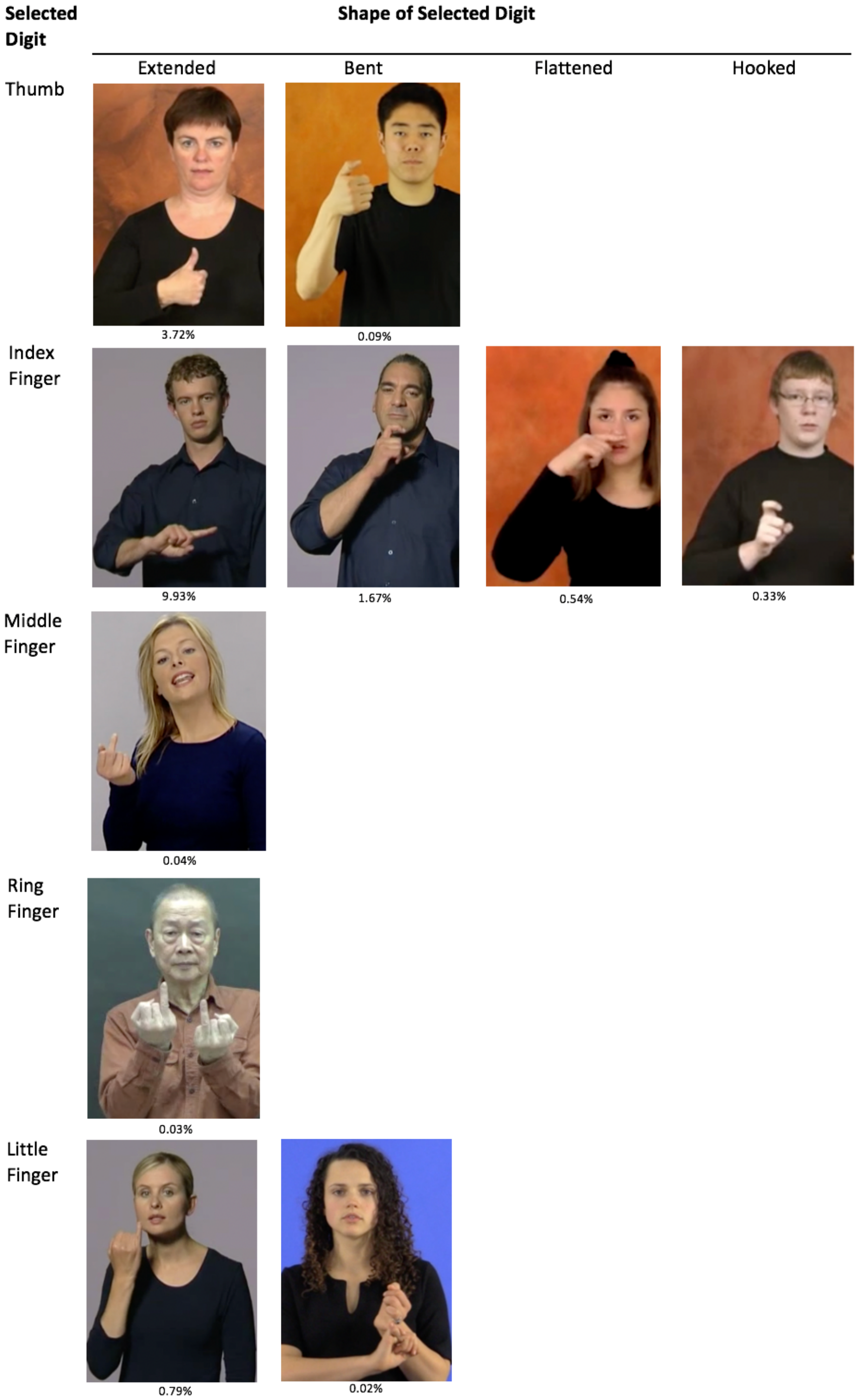

2.2 Non-selected digits are not closed

Only the index finger or the middle finger is selected in this type of handshapes. The index finger and the middle finger are bent or flattened. The non-selected fingers are all extended, but vary as to whether or not they are spread. The thumb is either extended or adducted.

2.2a Spread non-selected fingers

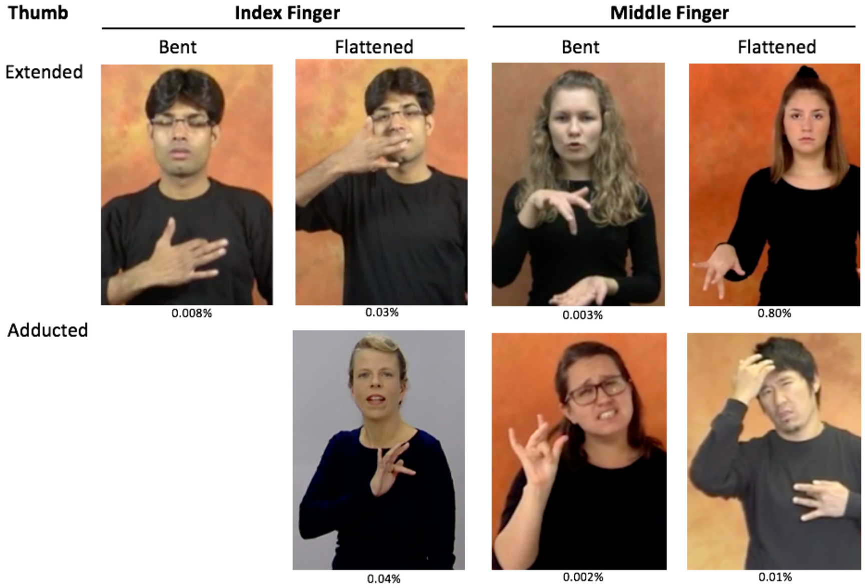

2.2b Non-selected fingers are not spread

In both of these handshapes, the selected finger – either the index finger (A) or the middle finger (B) – is bent. The thumb is adducted in A, extended in B.

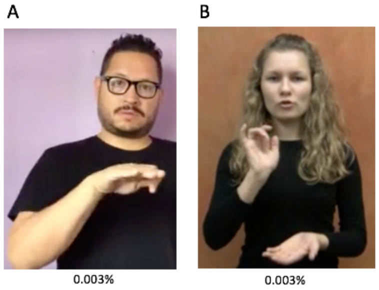

2.3 Complex handshapes

The selected finger in these handshapes is either the thumb, the index finger, or the little finger.

- Selected thumb. The thumb is bent, contrasting to the fingers that are extended.

- Selected index finger. The shape of the index finger varies in these handshapes – it is extended in A and B, bent in C. Non-selected fingers vary in shape as well – they are flattened in A and B, bent in C. The thumb is adducted in A. The fingertip of the thumb is opposed to the fingertips of the non-selected fingers in B and C.
- Selected little finger. The little finger is extended in both handshapes. The thumb makes contact with the non-selected fingers, which are bent in A, flattened in B.

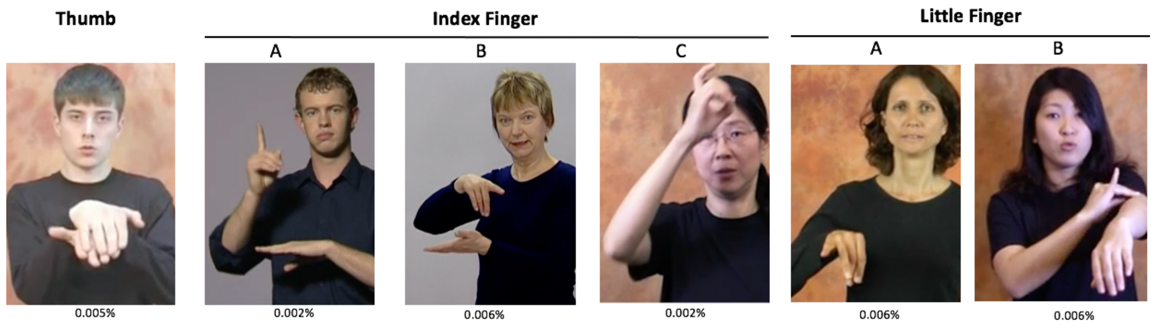

3 Selected finger and thumb

The index, middle, or little finger is selected along with the thumb. The selected finger and the thumb vary in this type of handshapes in shapes and the presence of thumb-finger opposition. The selected finger and thumb, which are closed, extended, or rounded, differ in shape from the non-selected fingers.

3.1. No thumb-finger opposition; closed non-selected fingers

3.1a. Selected index finger and thumb

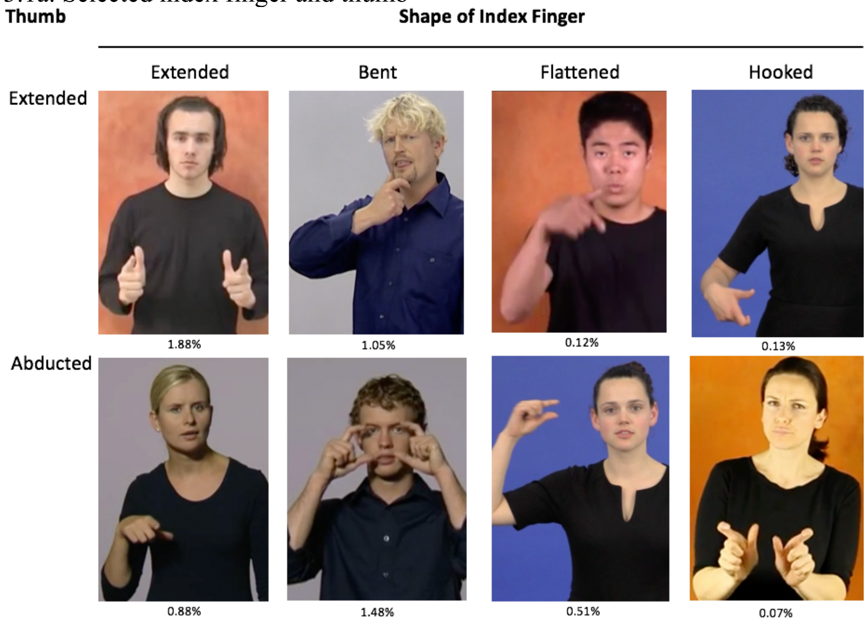

3.1b Selected index finger and thumb – complex handshapes

In A, the thumb is in between the index and the middle finger. In B and C, the index finger is bent; the thumb is abducted and contacts the index finger at the level of the middle phalanx (B) or of the carpal-metacarpal joint (C).

In D, the index finger is bent, the thumb is extended, and the middle, ring and little fingers are extended and not spread. In E, the index finger is bent and the thumb is abducted, forming a kind of C shape; the non-selected fingers are rounded.

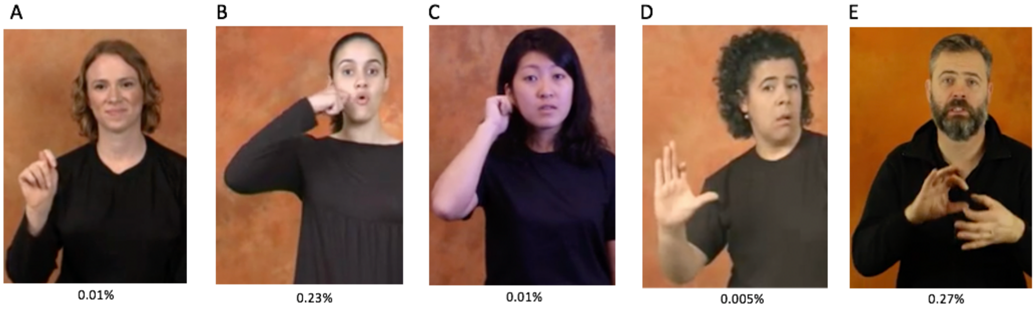

3.1c Selected middle finger and thumb

The thumb is extended; the middle finger is flattened (A) or extended (B).

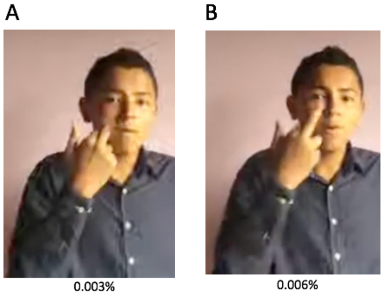

3.1d Selected little finger and thumb

The thumb and little finger are extended (A) or bent (B).

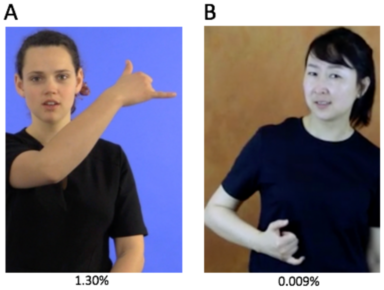

3.1e Selected little finger and thumb – complex handshape

The selected little finger and thumb are extended; the non-selected fingers are flattened.

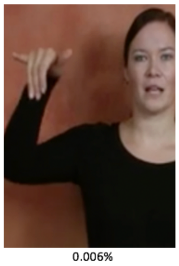

3.2 No thumb-finger opposition; extended non-selected fingers

The finger selected along with the thumb is either the index finger (A) or the middle finger (B and C). The selected finger is flattened (A and C) or bent (B). The thumb is abducted.

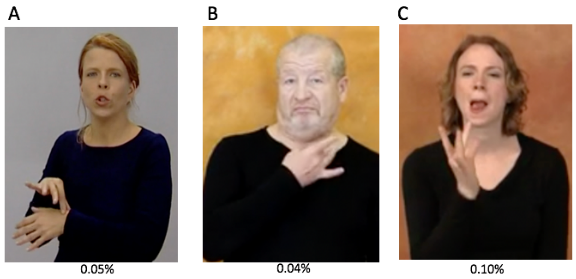

3.3 Thumb-finger opposition; closed non-selected fingers

The thumb and the index finger are selected, their tips opposed. The shape of the index finger varies – bent (A), flattened (B), or L-shaped (C).

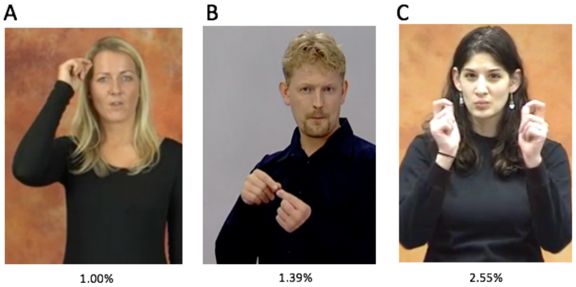

3.4 Thumb-finger opposition; extended non-selected fingers

The thumb is opposed to one of the fingers, contact occurs at tip point. The selected finger and thumb are either rounded or flattened. Non-selected fingers vary for being rounded (left and central columns) or flattened (right column).

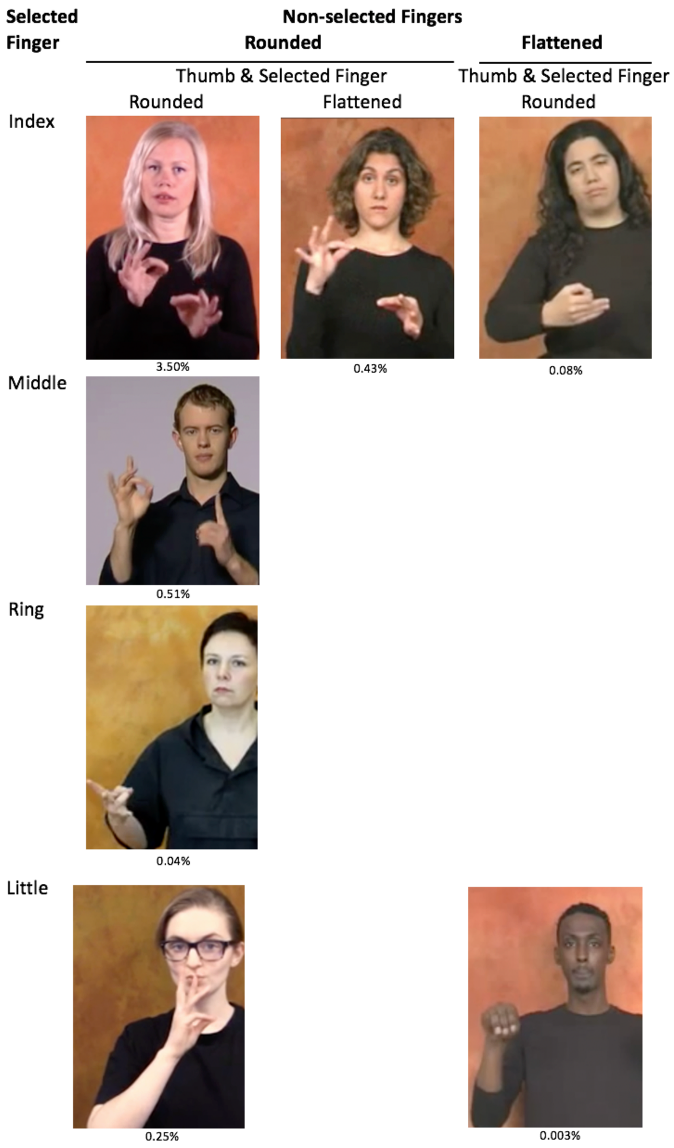

3.5 Thumb-index opposition – complex handshapes

The index makes contact with the thumb at different locations in these handshapes: the carpal-metacarpal joint connecting the thumb to the hand (A); the interphalangeal joint connecting the two parts of the thumb (B); the dorsal surface of the thumb, so that the index finger is above the thumb (C).

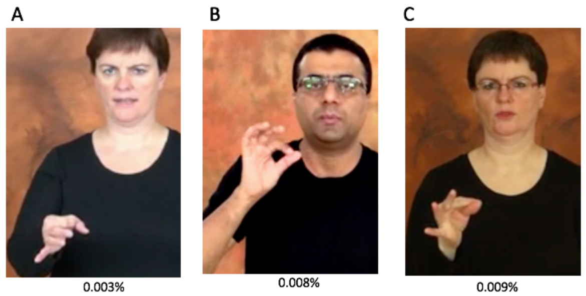

4 **Two fingers selected**
The selected fingers are the index and middle fingers (A), the index and little fingers (B), or the ring and little fingers (C).

4.1 Selected index and middle fingers
The index and middle fingers differ from the non-selected digits (thumb, ring and little fingers) that are closed. The two selected fingers vary depending on whether or not they are spread. They are shaped identically or differently, and their shapes vary (extended, bent, flattened, hooked).

4.1a Identically shaped index and middle fingers

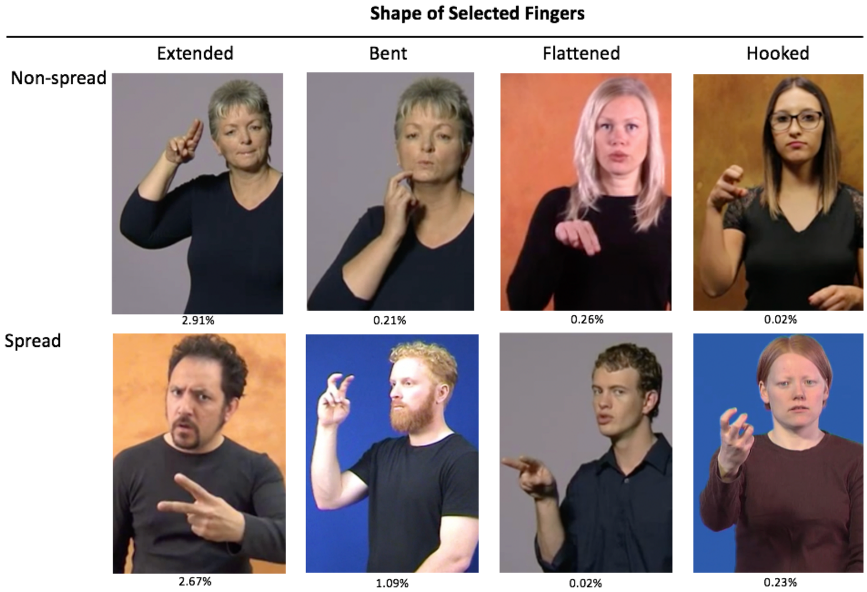

4.1b Differently shaped index and middle fingers

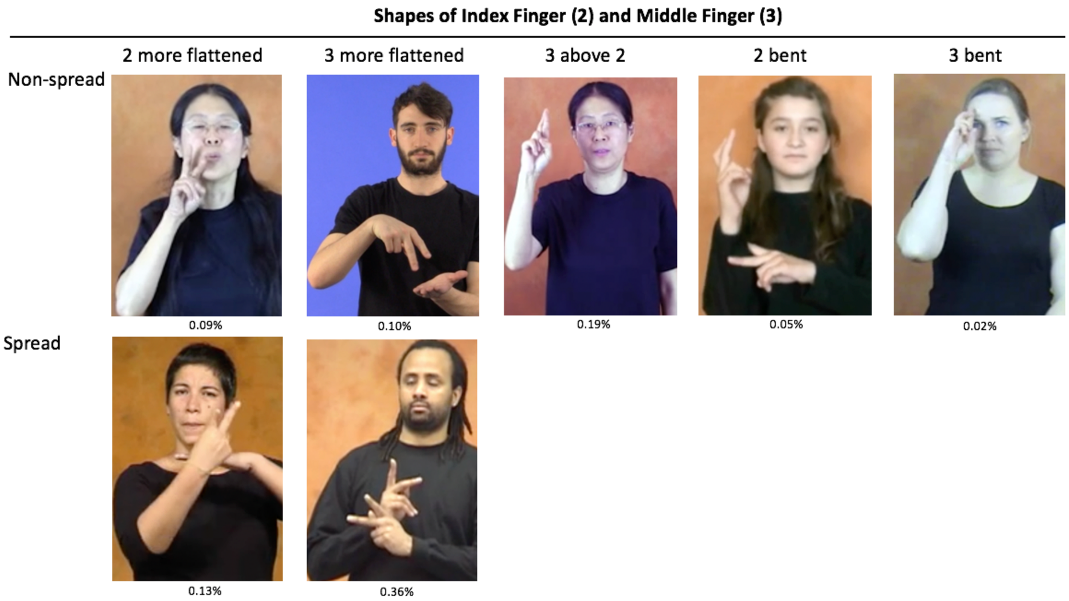

4.2 Selected index and little fingers

The selected index and little fingers are extended, in contrast to the non-selected fingers that are bent (A), flattened (B and D) or closed (C). Handshapes A and B vary with respect to thumb shape – extended vs. adducted, respectively. In C and D, the non-selected fingers (middle and ring fingers) are opposed to the thumb.

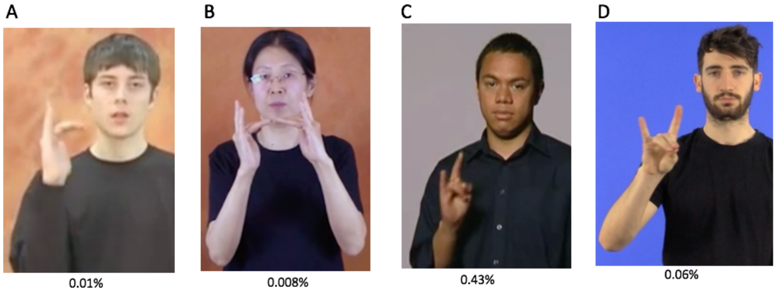

4.3 Selected ring and little fingers

The ring and little fingers are extended, in contrast to the non-selected fingers (index and middle fingers) that are closed (A) or flattened (B).

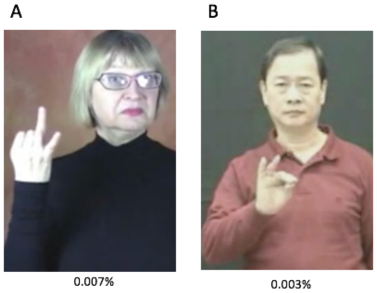

4.4 Complex handshapes

In A, the selected index and middle fingers are flattened – in contrast to the non-selected fingers that are closed – and the middle finger is above the index finger. In B, the selected middle and the ring fingers are flattened; the non-selected index and little fingers are extended.

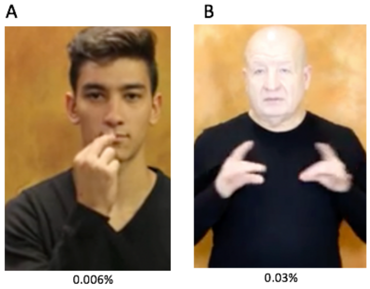

5. Two fingers and thumb selected

The index and middle fingers or the index and little fingers are selected along with the thumb. The two selected fingers and the thumb are differently shaped compared to the non-selected fingers that are closed or stacked.

5.1 Selected and identically shaped index and middle fingers

The index and middle fingers do not make contact with the thumb, and are either spread or not spread. This type of handshapes vary for the configuration of the index and middle fingers (extended, bent, flattened, or hooked) as well as for thumb position (extended or abducted).

5.1a Index and middle fingers are spread

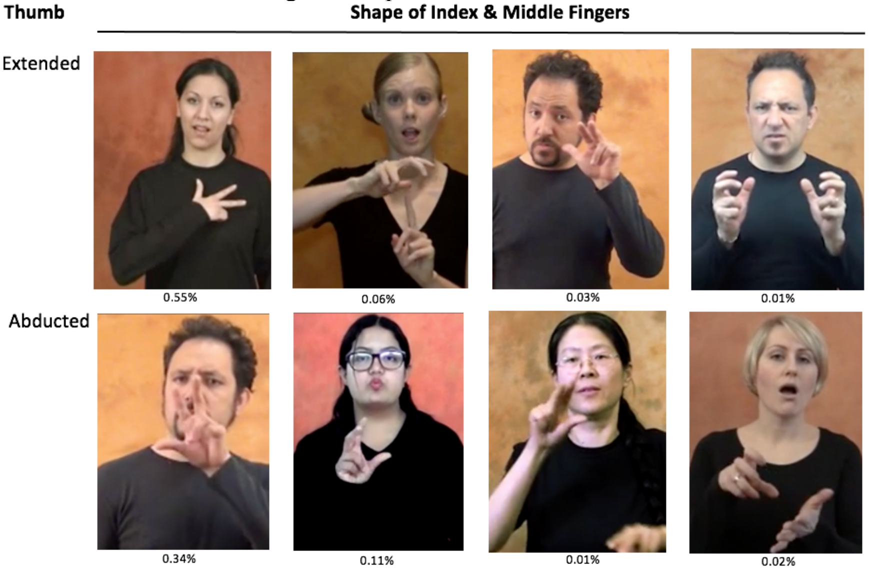

5.1b Index and middle fingers are not spread

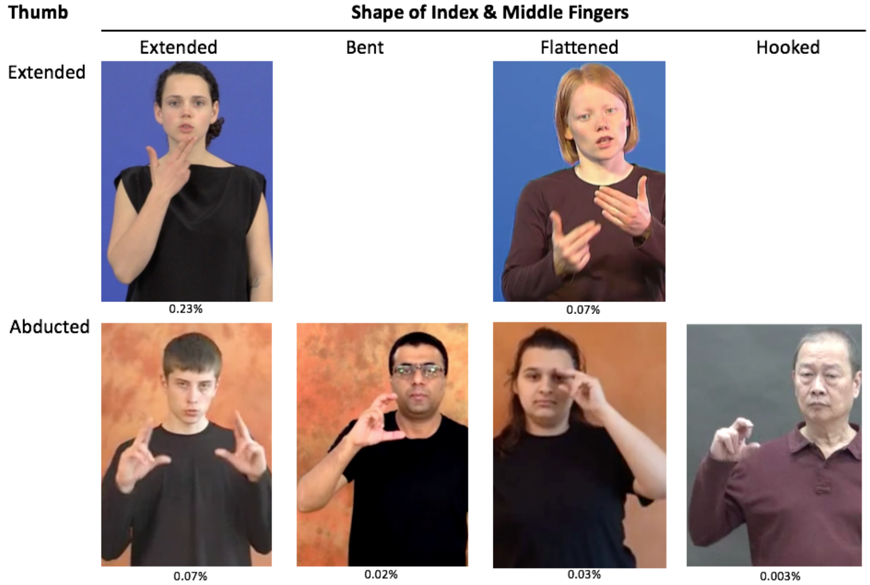

5.2 Selected and differently shaped index and middle fingers
The index and middle fingers do not make contact with the thumb, and are either spread or not spread. This type of handshapes vary for the configuration of the index and middle fingers as well as for thumb position (extended or abducted).

5.2a Index and middle fingers are spread

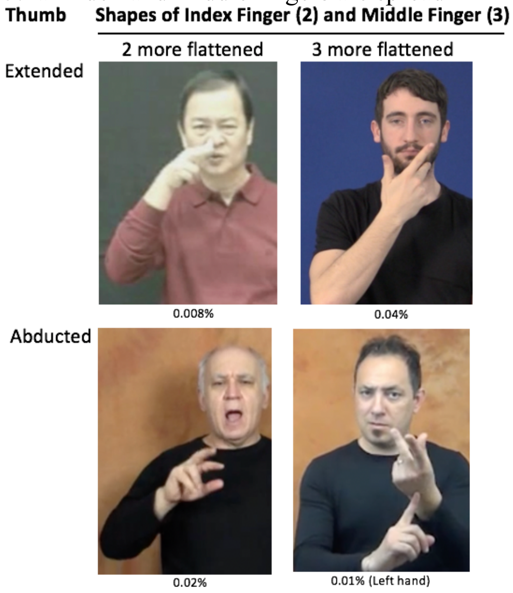

5.2b Index and middle fingers are not spread

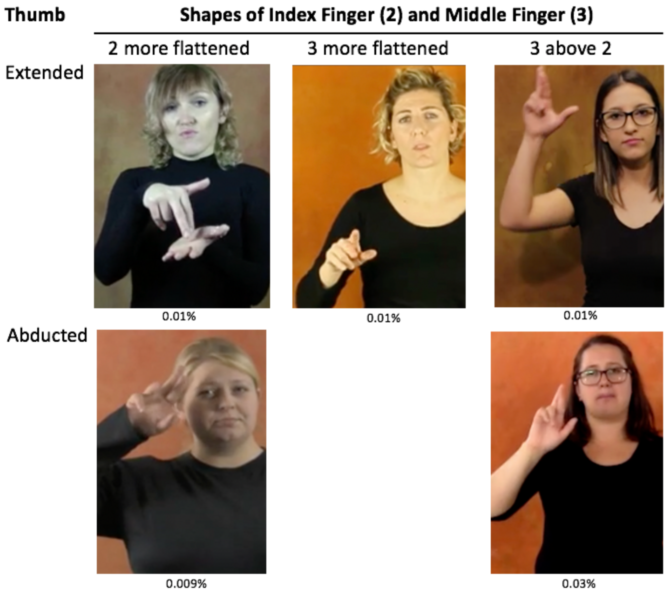

5.2c Complex handshapes
The thumb is extended and bent (A). The index and middle fingers are selected in handshapes in B-F. The index finger differs from the middle finger for being extended (B), bent (C), hooked (D). The middle finger is above the index finger (E and F); the index and middle fingers are bent (E) or flattened (F).

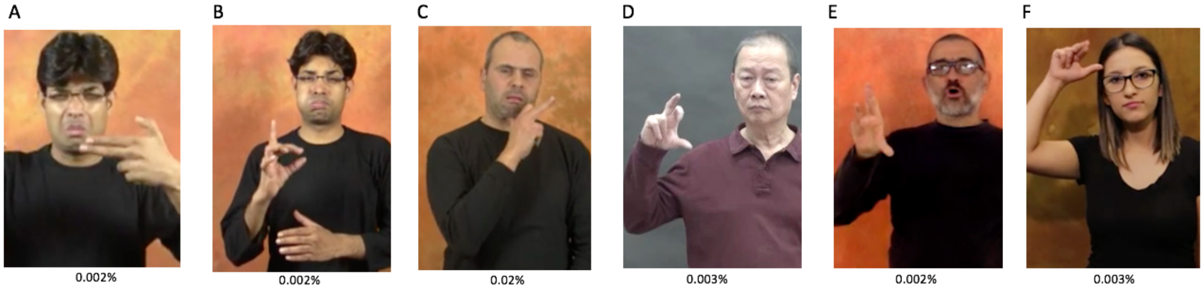

5.3 Fingertip-thumbtip opposition
The index and middle fingers are opposed to the thumb (A and B). The index and middle fingers are bent (A) or flattened (B). Only the middle finger is opposed to the thumb in C.

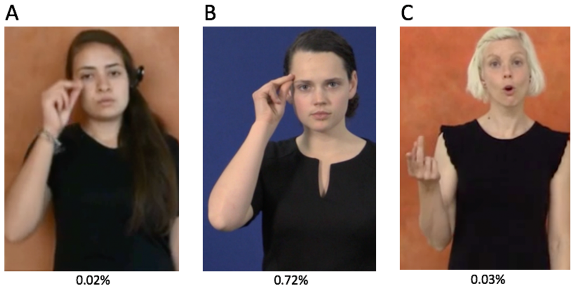

5.4. Complex handshapes

Only one selected finger is opposed to the thumb – the index finger in A, the middle finger in B and C. Th finger that is not opposed to the thumb varies in shape – extended (A and B), hooked (C).

A

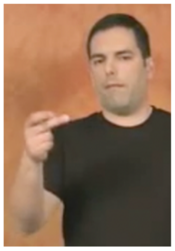

0.002%

B

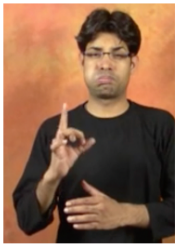

0.002%

C

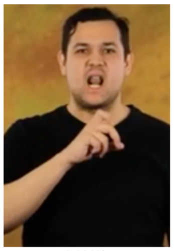

0.003%

5.5 Selected little finger

The little finger and the thumb are selected along with the index finger (A) or the ring finger (B).

A

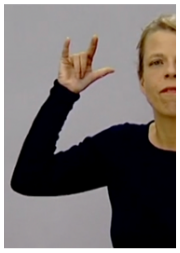

0.08%

B

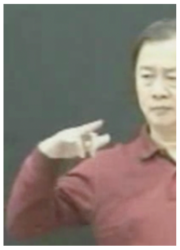

0.003%
